# WNT7B Promotes Bone Formation in part through mTORC1

**DOI:** 10.1371/journal.pgen.1004145

**Published:** 2014-01-30

**Authors:** Jianquan Chen, Xiaolin Tu, Emel Esen, Kyu Sang Joeng, Congxin Lin, Jeffrey M. Arbeit, Markus A. Rüegg, Michael N. Hall, Liang Ma, Fanxin Long

**Affiliations:** 1 Department of Orthopaedic Surgery, Washington University School of Medicine, St. Louis, Missouri, United States of America; 2 Department of Medicine, Washington University School of Medicine, St. Louis, Missouri, United States of America; 3 Division of Biology and Biomedical Sciences, Washington University in St. Louis, St. Louis, Missouri, United States of America; 4 Department of Surgery, Washington University School of Medicine, St. Louis, Missouri, United States of America; 5 Biozentrum, University of Basel, Basel, Switzerland; 6 Department of Developmental Biology, Washington University School of Medicine, St. Louis, Missouri, United States of America; Seattle Children's Research Institute, United States of America

## Abstract

WNT signaling has been implicated in both embryonic and postnatal bone formation. However, the pertinent WNT ligands and their downstream signaling mechanisms are not well understood. To investigate the osteogenic capacity of WNT7B and WNT5A, both normally expressed in the developing bone, we engineered mouse strains to express either protein in a Cre-dependent manner. Targeted induction of WNT7B, but not WNT5A, in the osteoblast lineage dramatically enhanced bone mass due to increased osteoblast number and activity; this phenotype began in the late-stage embryo and intensified postnatally. Similarly, postnatal induction of WNT7B in *Runx2*-lineage cells greatly stimulated bone formation. WNT7B activated mTORC1 through PI3K-AKT signaling. Genetic disruption of mTORC1 signaling by deleting *Raptor* in the osteoblast lineage alleviated the WNT7B-induced high-bone-mass phenotype. Thus, WNT7B promotes bone formation in part through mTORC1 activation.

## Author Summary

The human bone tissue is of considerable regenerative capacity as reflected in bone remodeling and in fracture healing. However, bone tissue regeneration deteriorates with age, and tremendous unmet medical needs exist for safe and effective strategies to stimulate bone formation in older individuals commonly inflicted with osteoporosis or osteopenia. WNT signaling has emerged as a promising target pathway for developing novel bone anabolic therapeutics. Identifying bone-promoting WNT ligands and elucidating the underlying mechanisms may lead to useful therapeutic targets. The present study reports that WNT7B potently enhances bone formation through activation of mTORC1 in the mouse.

## Introduction

WNT proteins are a family of signaling molecules that control cell proliferation, fate decision, polarity and migration throughout metazoan evolution [1]. By engaging various receptors and co-receptors at the cell membrane, these proteins activate a context-dependent intracellular signaling network to induce diverse biological responses [2]. Deregulation of WNT signaling is frequently associated with human diseases [3]. WNT signaling was first associated with bone diseases by the finding that loss-of-function mutations in the WNT co-receptor LRP5 cause osteoporosis-pseudoglioma syndrome (OPPG) (Gong et al., 2001). In contrast, deficiency in the secreted WNT inhibitor SOST, or mutations in LRP5 rendering it refractory to the WNT inhibitors such as SOST or DKK1, results in high bone mass in patients [4], [5], [6], [7], [8], [9]. In addition, mutations in WTX, an inhibitor of WNT/β-catenin signaling, were shown to cause X-linked sclerosing bone dysplasia known as OSCS in humans [10], [11]. In the mouse, deletion of LRP5 either globally or specifically in bone causes osteopenia in the mouse [12], [13], whereas expression of the high-bone-mass forms of LRP5 increases bone accrual [13], [14]. Moreover, mice lacking one *DKK1* allele or both *SOST* alleles exhibit a higher bone mass [15], [16]. Overall, genetic evidence from both humans and mice supports the importance of WNT signaling in bone formation.

The intracellular signaling network mediating WNT function in bone formation is not completely understood [17]. Work in the mouse embryo has shown that deletion of β-catenin, or both LRP5 and LRP6, in the osteogenic progenitors abolishes osteoblast differentiation, indicating that β-catenin is likely a critical component of the WNT signaling network responsible for embryonic osteoblastogenesis [18], [19], [20], [21], [22]. However, these mice die at birth, and therefore are not useful for assessing whether or not β-catenin similarly mediates WNT function in postnatal bone formation. We and others have recently shown that deletion of β-catenin in Osx-expressing cells selectively in postnatal mice reduced the life span and activity of osteoblasts, as well as increasing adipogenesis in the bone marrow [23], [24]. Besides β-catenin, activation of PKCδ or CAMKII by WNT through phosphatidylinositol signaling has also been shown to promote osteoblast differentiation [25], [26]. In addition, multiple WNT ligands have been reported to activate mTORC1 (mammalian target of rapamycin complex 1) [27], [28]. We have recently shown that WNT proteins also activate mTORC2 to stimulate glycolysis [29]. mTORC1 differs from mTORC2 in that it uniquely contains Raptor and is acutely sensitive to rapamycin [30]. Because mTORC1 signaling is a central mechanism integrating extracellular and intracellular cues with anabolic metabolism, it could potentially mediate WNT function during bone formation. Overall, WNT proteins may promote bone anabolism through a signaling network composed of multiple interconnecting modules.

Despite a clear role for WNT signaling, the physiological WNT ligands promoting bone formation are just beginning to be elucidated. WNT1 has recently been linked to bone physiology in humans, as heterozygous or homozygous mutations have been identified in patients with inherited early-onset osteoporosis or osteogenesis imperfecta, respectively [31], [32], [33], [34]. In the mouse, WNT10B has been implicated in postnatal bone formation [35], [36], but the low bone mass phenotype in the *Wnt10b^−/−^* mice appears later in life than the *Lrp5^−/−^* animals [37], indicating that LRP5 may interact with other WNT ligands at the earlier stages. In the mouse embryo, WNT7B is specifically expressed within the osteogenic perichondrium; deletion of *Wnt7b* in the skeletal osteoprogenitors causes a delay in ossification, but the phenotype is modest and largely resolved by birth, likely due to WNT ligand redundancy [18], [25]. In addition, WNT5A is expressed in both the perichondrium and the cartilage in the mouse embryo [18], [38]. Studies to date have indicated that WNT5A expressed by osteoblast-lineage cells promotes both osteoblastogenesis and osteoclastogenesis, but WNT5A deficiency causes a net decrease in bone mass in postnatal mice [26], [39].

In this study, we investigate the capacity of WNT7B versus WNT5A in regulating bone mass in vivo. We demonstrate that WNT7B dramatically enhances bone formation. Mechanistic studies identify mTORC1 as an important mediator for the bone anabolic function of WNT7B.

## Results

### WNT7B, but not WNT5A, increases bone mass *in vivo*

**Figure 1:**
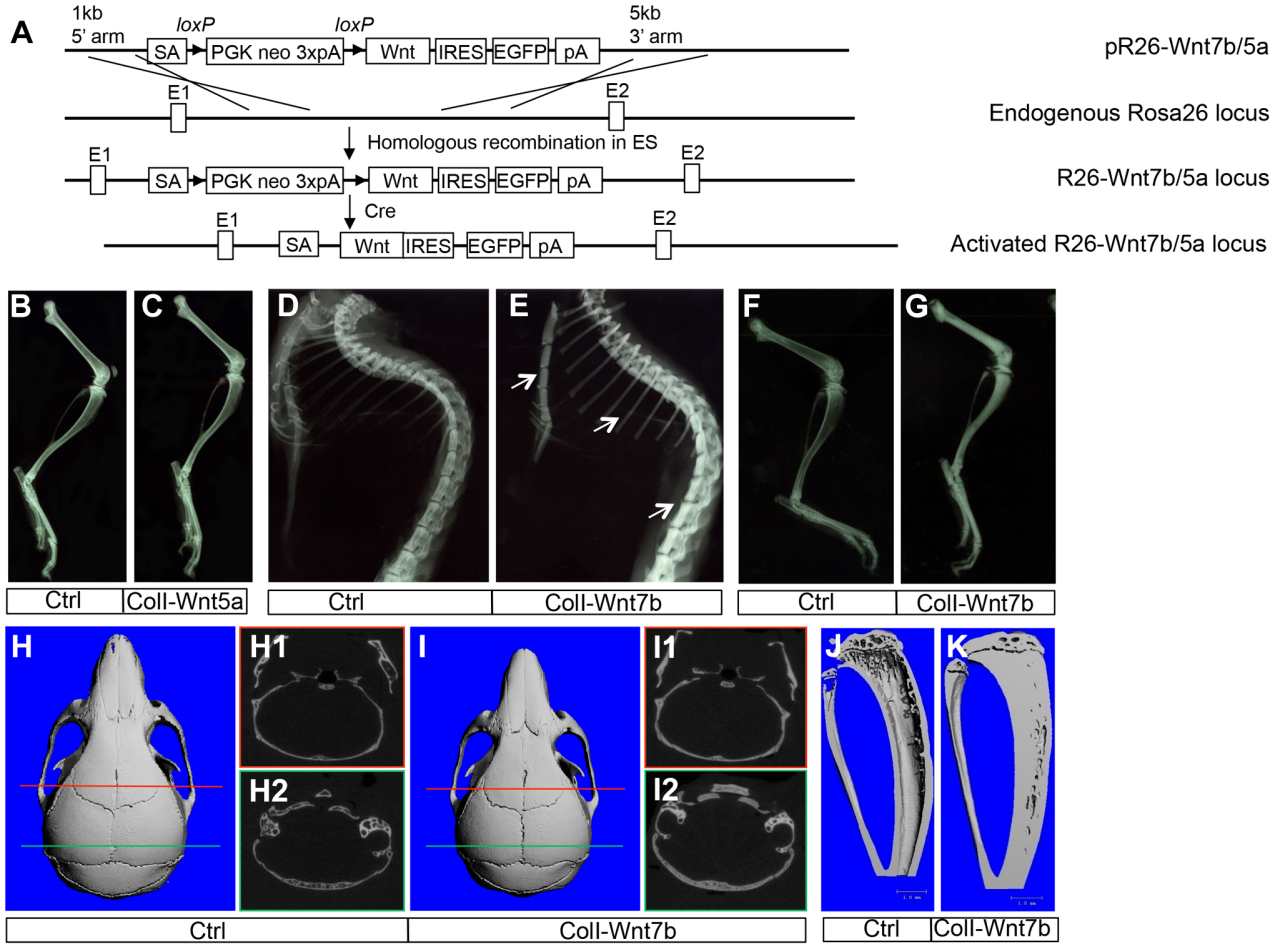
WNT7B, but not WNT5A, increases bone mass *in vivo*. (A) A schematic for generating mice with Cre-dependent overexpression of WNT7B or WNT5A. (B–C) X-ray images of hindlimbs of two-month-old *ColI-Cre* (Ctrl) (B) or *ColI-Wnt5a* littermate mice (C). (D–G) X-ray images of the axial skeleton (D, E) and hindlimbs (F, G) of two-month-old *ColI-Cre* (Ctrl) (D, F) versus *ColI-Wnt7b* littermate mice (E, G). Arrows denote increased mineral density in sterna, ribs and spine. (H–I) µCT 3D reconstruction of skulls from two-month-old *ColI-Cre* (Ctrl) (H) or *ColI-Wnt7b* littermate mice (I). H1, H2, I1, I2 show a single-slice µCT scan at positions indicated by the red or green line. (J, K) µCT 3D reconstruction of tibias from two-month-old *ColI-Cre* (Ctrl) (J) or *ColI-Wnt7b* littermate mice (K).

**Figure 2:**
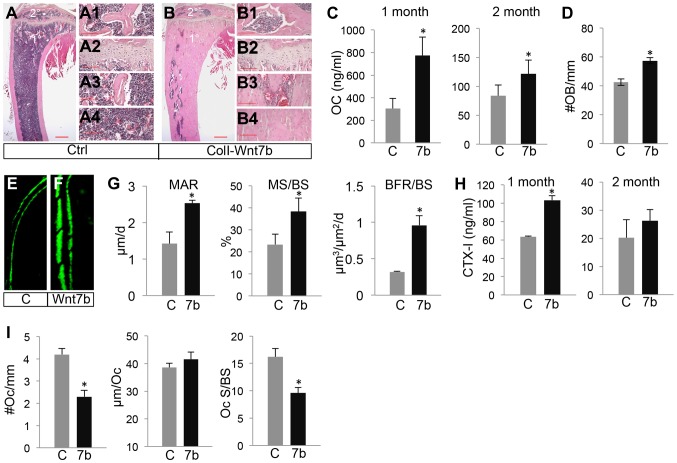
WNT7B increases osteoblast number and activity. (A, B) H&E staining of longitudinal tibia sections from two-month-old control (A) or *ColI-Wnt7b* littermates (B). 1°, 2°: primary and secondary ossification center. Shown to the right are higher magnification images of secondary ossification center (A1, B1), growth plate (A2, B2), primary spongiosa (A3, B3), and marrow region (A4, B4). Scale bar: 0.5 mm in panels A, B; 0.1 mm in panels A1–A4, B1–B4. (C) Serum osteocalcin levels of control (C) and *ColI-Wnt7b* littermates (7b) at one and two months of age. (D) Number of osteoblasts normalized to trabecular bone perimeter on longitudinal tibia sections. (E–F) Representative images of calcein double labeling in the humerus of two-month-old control (E) and *ColI-Wnt7b* (F) littermates. (G) Dynamic histomorphometry parameters from secondary ossification center of the humerus. MAR: mineral apposite rate; MS/BS: mineralizing surface over bone surface; BFR/BS: bone formation rate. (H) Serum CTX-I levels. (I) Osteoclast parameters from histomorphometry. #Oc/mm: osteoclast number normalized to trabecular bone perimeter, µm/Oc (average osteoclast surface), Oc S/BS (osteoclast surface normalized to bone surface). All bar graphs show mean ± STDEV, *: P<0.05, n = 3.

To study the roles of WNT7B and WNT5A, we first developed versatile mouse strains that allow these proteins to be expressed in a tissue-specific manner. Specifically, we knocked the *Wnt7b* or *Wnt5a* cDNA into the ubiquitously active Rosa26 locus so that they can be expressed upon the excision of a transcriptional stop signal by Cre (Fig. 1A) (Fig. S1). The resultant mouse strains, *R26-Wnt7b* or *R26-Wnt5a*, did not show any discernible phenotype in either heterozygous or homozygous state. To assess the potential role of either protein in bone formation, we activated their expression in the osteoblast lineage with either *Osx-Cre* targeting preosteoblasts or *2.3ColI-Cre* targeting the more mature osteoblast-lineage cells. Mice expressing WNT5A from one or two *R26-Wnt5a* alleles by *2.3Col1-Cre* appeared normal, and did not exhibit any obvious bone phenotype when analyzed by X-ray radiography or µCT at two months of age (Fig. 1B, C) (Table 1). The *R26-Wnt5a* allele was functional because its activation with *Wnt1-Cre* in neural crest cells caused embryonic lethality with multiple cranial facial defects (data not shown). We therefore focused on WNT7B in the remainder of the study. Mice with WNT7B expression from a single *R26-Wnt7b* allele by either *Osx-Cre* or *2.3ColI-Cre* (hereafter *Osx-Wnt7b* or *ColI-Wnt7b* mice) were viable without any gross abnormality. However, X-ray radiography of either mutant at two months of age detected profoundly dense bones throughout the body (Fig. 1D–G) (Fig. S2). The X-ray images also revealed shorter bones in the *Osx-Wnt7b* mice when compared to their control littermates. The mechanism for the size difference was not investigated in the present study, but to avoid size-related complications we have focused the postnatal analyses on the *ColI-Wnt7b* mice with a normal bone size. The severity of the high-bone-mass phenotype in *ColI-Wnt7b* mice was epitomized by the lack of marrow space in the long bones due to complete ossification (Fig. 1G). As expected, these mice exhibited splenomegaly consistent with extramedullary hematopoiesis (Fig. S3A–C). The high-bone-mass phenotype was fully penetrant in both males and females, and persisted at six months of age but was partially resolved at nine months (Fig. S4A, B). The mechanism for the phenotype amelioration with aging was not fully pursued here, but appeared to track with heightened bone resorption, as indicated by the higher serum CTX-I level (C-terminal telopeptide of type I collagen, a degradation product of type I collagen released upon bone resorption) than the control, at nine but not six months of age (Fig. S4C, D). MicroCT analyses of the two-month-old *ColI-Wnt7b* mice confirmed the profound high-bone-mass phenotype in both the skull and long bones (Fig. 1H–J). The proximal tibial trabecular BV/TV was 13.7-fold elevated compared to control at two months, coupled with increased trabecular thickness and reduced trabecular spacing (Table 2). At six months of age, BV/TV in the same area was 5.1 fold higher in *ColI-Wnt7b* mice than the littermate control. Consistent with X-ray radiography, by nine months, the high bone mass in the proximal tibial trabecular area was resolved and in fact 30% less than the littermate control, even though the distal tibia and the femur maintained a high bone mass (Fig. S4B) (Table 2). Histology confirmed that excessive bone occupied both primary and secondary ossification centers, whereas the growth plate was largely normal in the *ColI-Wnt7b* mice (Fig. 2A, B). Thus, WNT7B induction in osteoblast-lineage cells markedly increases bone mass throughout the body in postnatal mice.

**Table 1:** µCT analyses of ColI-Wnt5a at 2 months of age.

Mouse	BV/TV			Tb. N*			Tb. Th*			Tb. Sp*		
	(%)	Ratio	t-test	(1/mm)	Ratio	t-test	(mm)	Ratio	t-test	(mm)	Ratio	t-test
ColI-Wnt5a	6.87±0.19	**1.03**	P = 0.89	2.07±0.07	**0.93**	P = 0.25	0.057±0.001	**1.01**	P = 0.85	0.48±0.02	**1.04**	P = 0.30
WT	6.66±2.52			2.21±0.16			0.056±0.008			0.46±0.03		

BV: bone volume; TV; total volume; Tb. N*: trabeculae number; Tb. Th*: trabeculae thickness; Tb. Sp*: trabeculae spacing; data obtained from 100 of 16-µm slices immediately below growth plate, n = 3 for each group.

**Table 2:** µCT analyses of ColI-Wnt7b at 2, 6, and 9 months of age.

Age	Mouse	BV/TV			Tb. N*			Tb. Th*			Tb. Sp*		
		(%)	Ratio	t-test	(1/mm)	Ratio	t-test	(mm)	Ratio	t-test	(mm)	Ratio	t-test
2 months	Col-Wnt7b	99.64±0.29	**13.7**	P = 1.19E-07	3.56±0.48	**1.6**	P = 0.06	0.518±0.023	**9.0**	P = 0.0008	0.04±0.02	**0.8**	P = 0.01
	WT	7.23±1.88			2.19±0.82			0.057±0.003			0.51±0.18		
6 months	Col-Wnt7b	73.03±13.92	**5.1**	P = 0.001	3.21±0.45	**1.9**	P = 0.004	0.284±0.105	**3.5**	P = 0.028	0.34±0.09	**0.5**	P = 0.008
	WT	14.21±2.39			1.68±0.12			0.081±0.001			0.63±0.03		
9 months	Col-Wnt7b	11.73±1.87	**0.7**	P = 0.012	1.59±0.39	**0.6**	P = 0.119	0.088±0.007	**1.0**	P = 0.48	0.69±0.19	**0.8**	P = 0.12
	WT	17.74±1.54			2.52±0.71			0.084±0.005			0.43±0.13		

BV: bone volume; TV; total volume; Tb. N*: trabeculae number; Tb. Th*: trabeculae thickness; Tb. Sp*: trabeculae spacing; data obtained from 100 of 16-µm slices immediately below growth plate, n = 3 for each group.

### WNT7B increases osteoblast number and activity

We next investigated whether WNT7B increased bone mass by altering bone formation or resorption. To assess bone formation activity, we first measured serum levels of osteocalcin, a major non-collagenous protein produced by osteoblasts. Osteocalcin levels were significantly higher in *ColI-Wnt7b* than the control at both one and two months of age (Fig. 2C). Histomorphometry showed a higher osteoblast number normalized to bone surface in *ColI-Wnt7b* over control mice at two months of age (Fig. 2D). The density of osteocytes however was not changed (Fig. S5). Dynamic histomorphometry in these animals revealed that mineral apposition rate (MAR), the percentage of mineralizing surface (MS/BS), and bone formation rate (BFR/BS) were all increased in the humerus of *ColI-Wnt7b* over the normal counterpart (Fig. 2E–G). To examine whether WNT7B affected bone resorption, we measured serum CTX-I levels. Despite the high bone mass, *ColI-Wnt7b* mice exhibited a higher serum CTX-I level than normal at one month of age (Fig. 2H, left). At two months, CTX-I levels were similar between *ColI-Wnt7b* and control mice (Fig. 2H, right). Static histomorphometry showed that both osteoclast number per bone surface (#Oc/mm) and the percentage of bone resorption surface (Oc S/BS) were reduced in the *ColI-Wnt7b* mice at two months of age, whereas osteoclast spreading (µm/Oc) was not changed (Fig. 2I). These results indicate that osteoclastogenesis was likely suppressed in the WNT7B-overexpressing mice, but the total activity of bone resorption was not reduced at any of the ages examined. Thus, WNT7B increases bone mass mainly through stimulation of osteoblast number and activity.

### WNT7B stimulates bone acquisition in the embryo

**Figure 3:**
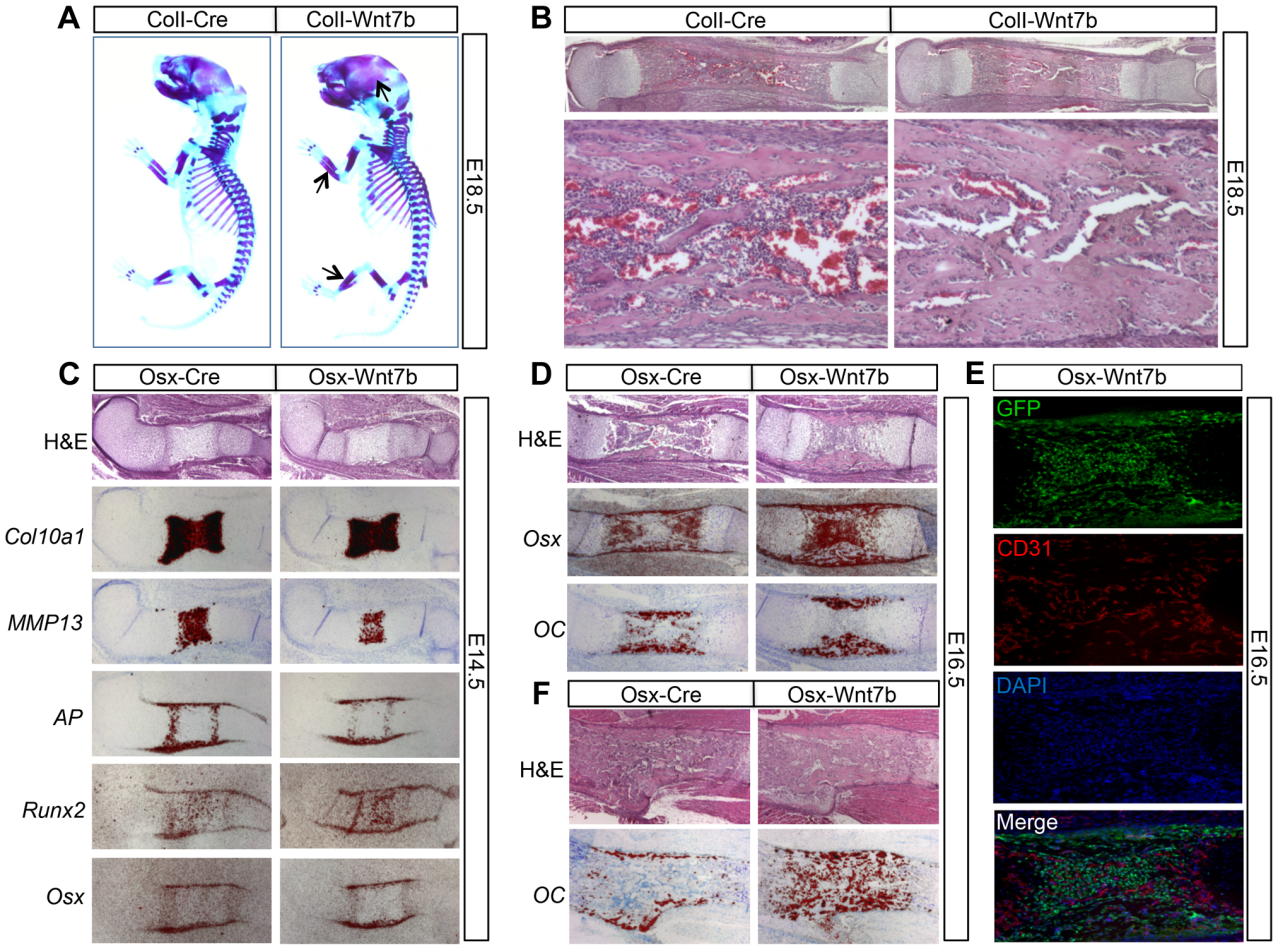
WNT7B stimulates bone acquisition in the embryo. (A) Whole-mount skeletal staining at E18.5. Arrows denote more bone in skull and limbs of *ColI-Wnt7b* embryos. (B) H&E staining of longitudinal tibial sections at E18.5. Shown below are images of the diaphyseal region at a higher magnification. (C, D) Analyses of longitudinal sections of the humerus at E14.5 (C) and E16.5 (D) by histology and *in situ* hybridization. (E) Immunostaining of GFP and CD31 on longitudinal sections of the humerus in E16.5 *Osx-Wnt7b* embryos. GFP: green; CD31: red; DAPI: blue. (F) Analyses of longitudinal sections of the humerus at E18.5 by histology and *in situ* hybridization. *In situ* hybridization signals shown in red.

As WNT7B induction by *2.3ColI-Cre* or *Osx-Cre* began in the embryo, we next determined whether WNT7B affected embryonic bone formation. Whole-mount skeletal staining with alcian blue and alizarin red revealed that at E18.5, both *ColI-Wnt7b* and *Osx-Wnt7b* embryos exhibited thicker bones with more intense red staining than normal, indicative of higher bone mass (Fig. 3A, data not shown). Histological sections of the embryonic long bones confirmed excessive bone mass occluding the presumptive marrow cavity (Fig. 3B, data not shown). Because both types of mutant embryos exhibited essentially the same phenotype, we have used either mutant for the embryonic analyses depending on their availability at the time of experiment. *In situ* hybridization of osteoblast markers in the bones of E18.5 *ColI-Wnt7b* embryos confirmed the presence of excessive osteoblasts within the presumptive marrow cavity (Fig. S6). Analyses of E14.5 *Osx-Wnt7b* embryos revealed a slight delay in chondrocyte maturation, as indicated by the shorter domains of *Col10a1* (general hypertrophy marker) and *MMP13* (late hypertrophy marker) (Fig. 3C). However, osteoblast differentiation in these embryos appeared to be normal, even though the expression domains of *AP*, *Runx2*, and *Osx* in the perichondrium were slightly reduced, as expected from the delay in chondrocyte maturation (Fig. 3C). At E16.5, the *Osx-Wnt7b* long bones possessed a much thicker bone collar than normal, but no bone marrow in stark contrast to the control (Fig. 3D). *In situ* hybridization revealed that the presumptive marrow region was occupied by cells expressing *Osx* but not *osteocalcin* (*OC*) and therefore likely to be preosteoblasts (Fig. 3D). Immunostaining for the endothelial marker CD31 indicated that the region was vascularized even though no marrow cavity was formed (Fig. 3E). At E18.5, the presumptive marrow region was populated with mature osteoblasts expressing *OC* (Fig. 3F). In summary, WNT7B does not prematurely initiate bone formation in the perichondrium, but augments the process in both cortical and trabecular regions of the late-stage embryo.

### WNT7B enhances bone accrual in postnatal mice

**Figure 4:**
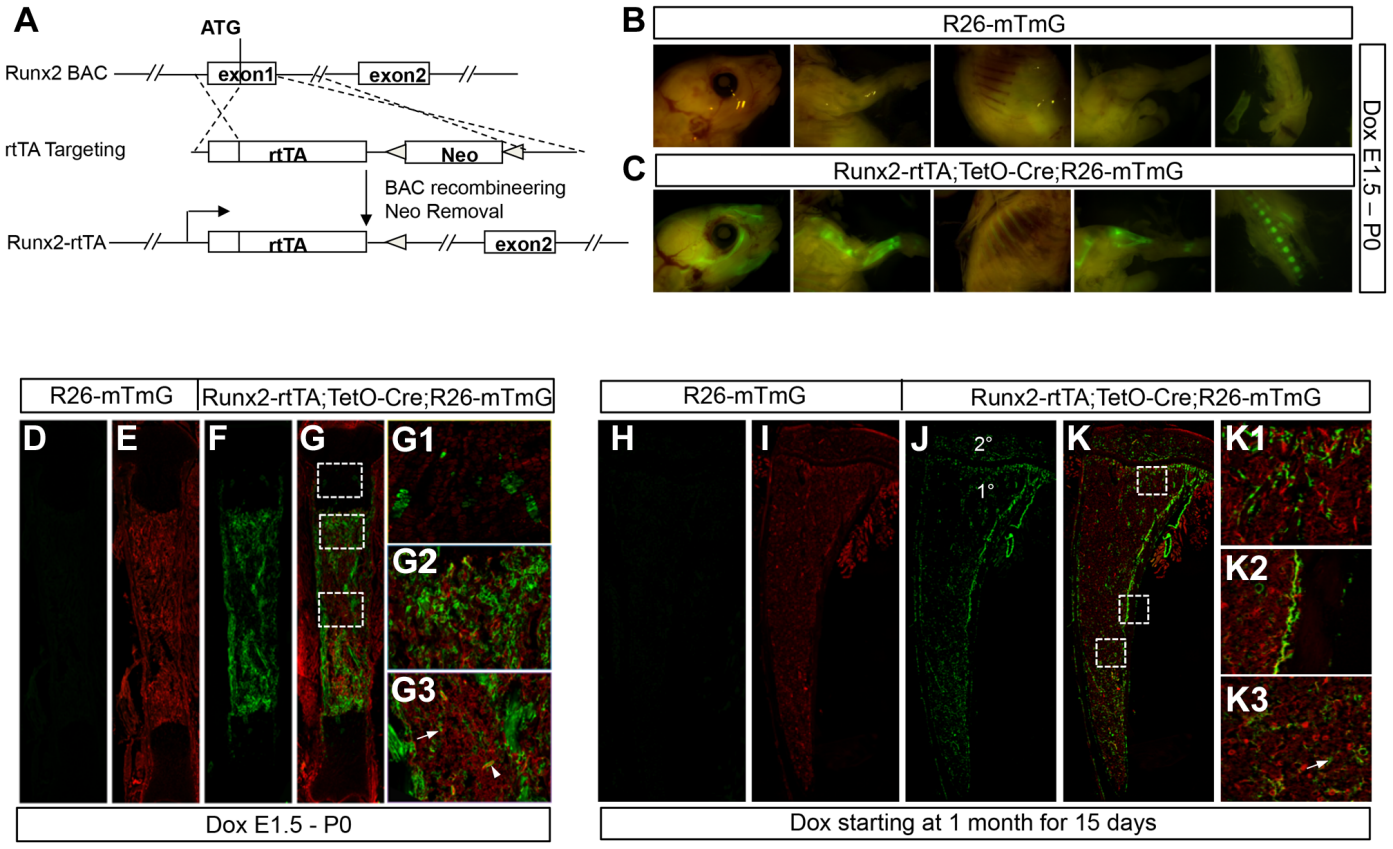
Generation and characterization of *Runx2-rtTA* transgenic mice. (A) A schematic for generating the *Runx2-rtTA* BAC transgenic mouse. (B–C) GFP imaging by fluorescence microscopy of whole-mount skeletal elements (left to right: skull, forelimb, ribs, hindlimb, vertebrae) from *R26-mTmG* (B) or *Runx2-rtTA;TetO-cre;R26-mTmG* (C) neonates treated with 1 mg/ml Dox in drinking water from E1.5 till birth. (D–G) Fluorescence imaging of frozen sections of the tibia from *R26-mTmG* (D, E) or *Runx2-rtTA; TetO-Cre; R26-mTmG* (F, G) neonates treated with 1 mg/ml Dox from E1.5 to birth. D, F: GFP single channel; E, G: GFP and RFP merged image. Boxed areas in G are shown at a higher magnification in G1 (growth plate), G2 (primary spongiosa) and G3 (diaphysis). (H–K) GFP detection on longitudinal tibial sections of *R26-mTmG* (H, I) or *Runx2-rtTA;TetO-Cre;R26-mTmG* (J,K) mice treated with 1 mg/ml Dox in drinking water for 15 days starting at 1 month of age. H, J: GFP immunofluorescence; I, K: merged images of GFP and RFP signals. RFP from direct fluorescence microscopy. Boxed areas in K are shown in K1 (primary spongiosa), K2 (cortical bones) and K3 (diaphyseal bone marrow). 1°, 2°: primary and secondary ossification center, respectively. Arrow: GFP^+^ bone marrow stromal cell; arrowhead: GFP^+^ perivascular cells.

We next sought to determine whether temporal activation of WNT7B specifically in postnatal bones stimulates bone formation. To this end, we created a mouse line (referred as *Runx2-rtTA*) that expressed reverse tetracycline transactivator (rtTA) from the *Runx2* regulatory elements through bacterial artificial chromosome (BAC) recombineering (Fig. 4A). To characterize the *Runx2-rtTA* line, we produced mice with the genotype of *Runx2-rtTA;TetO-Cre;R26-mT/mG* (termed *Runx2-mTmG*) and assessed GFP expression with or without doxycycline (Dox). Without Dox, no GFP was detected in these mice at either embryonic or postnatal stage (data not shown). When Dox was administered to the embryos through the dams, the *Runx2-mTmG* neonates, but not the control littermates, displayed strong GFP throughout the skeleton when viewed whole-mount under a fluorescence microscope (Fig. 4B, C). Confocal microscopy of long bone sections confirmed GFP expression only in the *Runx2-mTmG* neonates, but not in the control littermates (Fig. 4D–G). Closer examination of the *Runx2-mTmG* samples revealed GFP expression by a small subset of chondrocytes within the growth plate (Fig. 4G1), but most prominently in osteoblast-lineage cells associated with the primary spongiosa and the cortical bone (Fig. 4G2, G3). Additionally, GFP was detected in sporadic bone marrow stromal cells and perivascular cells located within the marrow cavity (Fig. 4G3). To characterize the *Runx2-rtTA* transgene postnatally, we raised the *Runx2-mTmG* mice until one month of age before treating them with Dox for 15 days. Whereas the control littermates exhibited no GFP (Fig. 4H, I), the *Runx2-mTmG* mice displayed GFP in both primary and secondary ossification centers as well as the cortical bone (Fig. 4J, K). Higher-magnification images revealed that GFP was expressed by cells associated with the trabecular bone within the primary and secondary ossification centers, the cortical bone, as well as by the marrow stromal cells, but not by growth plate chondrocytes (Fig. 4K1–K3) (Fig. S7). Staining for alkaline phosphatase activity revealed that the GFP-positive cells on the bone surfaces expressed the enzyme and therefore were most likely osteoblast-lineage cells (Fig. S8). Overall, the *Runx2-rtTA* mouse line provides a useful tool for targeting the osteoblast-lineage cells in postnatal animals.

**Figure 5:**
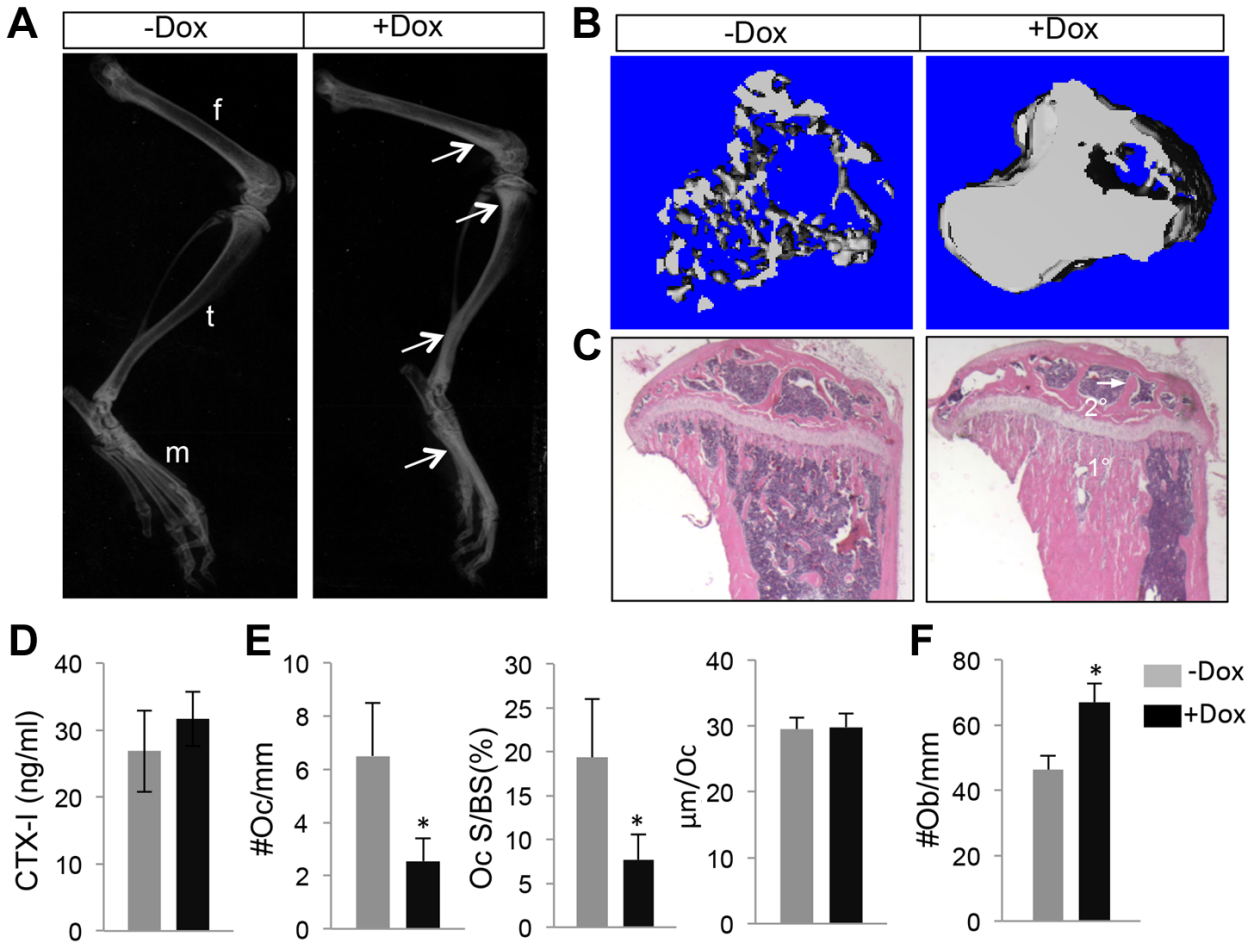
WNT7B enhances bone accrual in postnatal life. All data from *Runx2-rtTA;TetO-Cre;R26-Wnt7b* mice treated with (+Dox) or without (−Dox) 1 mg/ml Dox in drinking water from one month through two months of age. (A) X-ray images of hindlimbs. Arrows point to the places with increased bone mineral density. (B) µCT 3D reconstruction of metaphyseal trabecular bone of the tibia. (C) H&E staining of sections of the proximal tibias. (D) Serum CTX-I levels of two-month-old mice. (E) Histomorphometric parameters of osteoclasts on tibial sections. #Oc/mm: osteoclast number normalized to trabecular bone perimeter; Oc S/BS: osteoclast surface normalized to bone surface; µm/Oc: average osteoclast surface. (F) Number of osteoblasts normalized to trabecular bone perimeter on tibia sections. Bar graphs show mean ± STDEV, *: P<0.05, n = 3. f: femur; t: tibia; m: metatarsal.

We next employed the *Runx2-rtTA* allele to activate WNT7B expression in postnatal bones. Specifically, we generated mice with the genotype of *Runx2-rtTA;TetO-Cre;R26-Wnt7b* (hereafter *Runx2-Wnt7b*) and treated them with Dox from one month through two months of age. Untreated *Runx2-Wnt7b* mice did not have a phenotype compared to wild type littermates. Moreover, Dox itself did not affect bone mass in any of the control genotypes (missing at least one of the three alleles present in the *Runx2-Wnt7b* mouse). However, Dox notably increased bone mineral density in the long bones of *Runx2-Wnt7b* mice, as indicated by X-ray radiography (Fig. 5A). MicroCT analyses of the proximal tibial metaphysis revealed a 6.6-fold increase in trabecular BV/TV over the untreated littermates with the same genotype (Fig. 5B) (Table 3). Histology confirmed a marked increase in the trabecular bone mass in both primary and secondary ossification centers of the Dox-treated *Runx2-Wnt7b* mice (Fig. 5C). The increased bone mass was not produced by suppression of bone resorption, as serum CTX-I levels were unaltered in the Dox-treated mice (Fig. 5D), even though osteoclast number or surface normalized to bone surface was reduced (Fig. 5E). On the other hand, osteoblast numbers normalized to bone surface were markedly increased in the Dox-treated over non-treated *Runx2-Wnt7b* mice (Fig. 5F). Thus, temporal induction of WNT7B in postnatal mice greatly increases bone mass thorough stimulation of bone formation.

**Table 3:** µCT analyses of Runx2-Wnt7b with or without Dox from one through two months of age.

Mouse	BV/TV			Tb. N*			Tb. Th*			Tb. Sp*		
	(%)	Ratio	t-test	(1/mm)	Ratio	t-test	(mm)	Ratio	t-test	(mm)	Ratio	t-test
+Dox	61.59±11.51	**6.6**	P = 0.001	3.42±0.57	**1.3**	P = 0.16	0.232±0.038	**4.1**	P = 7.32E-06	0.37±0.03	**0.88**	P = 0.71
−Dox	9.28±3.43			2.54±0.76			0.057±0.004			0.42±0.10		

BV: bone volume; TV; total volume; Tb. N*: trabeculae number; Tb. Th*: trabeculae thickness; Tb. Sp*: trabeculae spacing; data obtained from 100 of 16-µm slices immediately below growth plate, n = 3 for each group.

### WNT7B and WNT3A activate mTORC1 signaling

**Figure 6:**
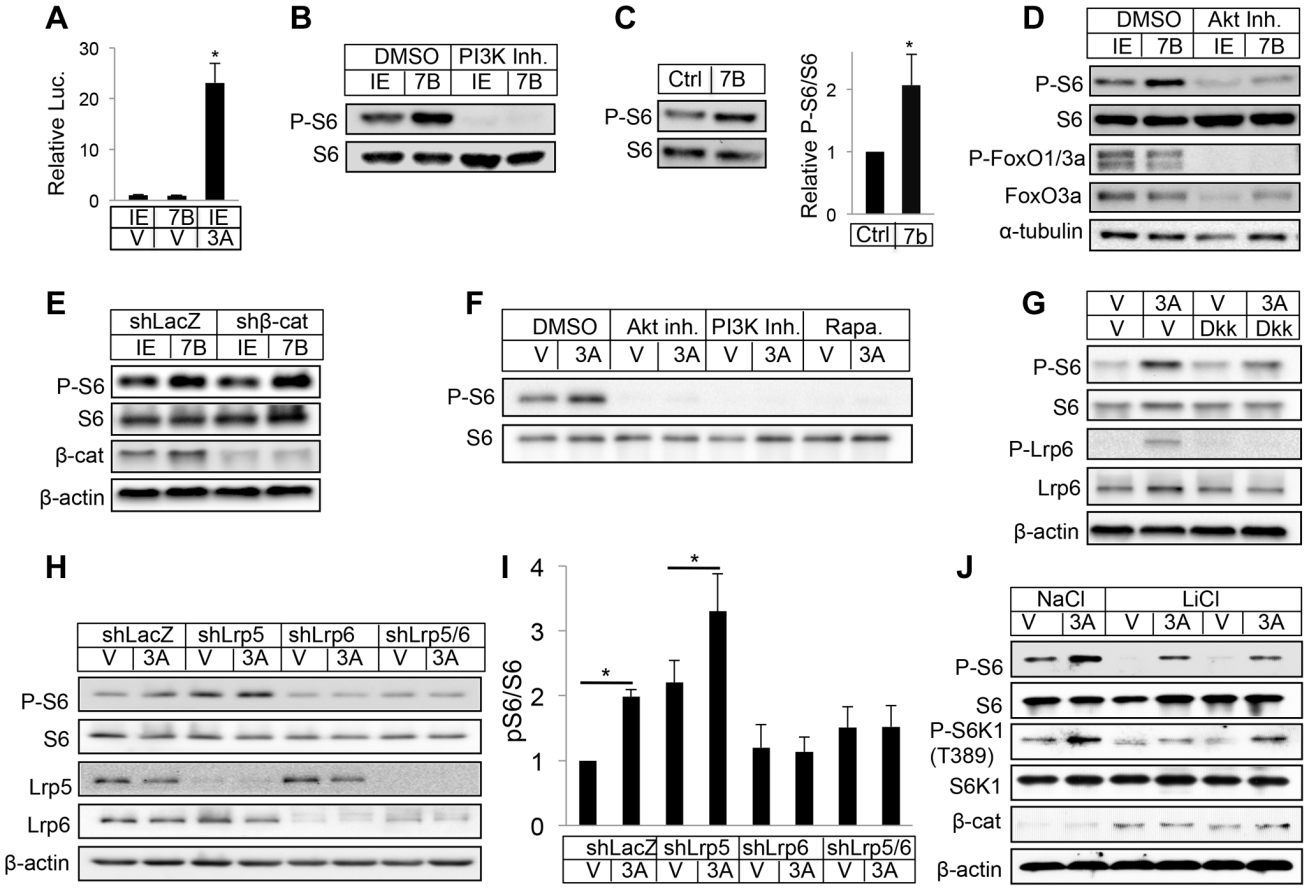
WNT7B and WNT3A activate mTORC1 signaling. (A) Transient transfection assays with luciferase reporter *Lef1-luc* in ST2 cells. IE: GFP-expressing control retrovirus; 7B: WNT7B-expressing retrovirus; V: vehicle (0.1% CHAPS in PBS); 3A: WNT3A. (B) Western blot with whole-cell lysates from ST2 cells infected with WNT7B or control (IE) retrovirus. Cells were serum-starved for 16 hours and then treated with inhibitor or vehicle for 2 hours before harvest. (C) Representative image (left) and quantification (right) of Western analyses with bone protein extracts from two-month-old *Osx-Cre* (Ctrl) and *Osx-Wnt7b* (7B) littermate mice. Levels of P-S6/S6 in control littermates designated 1. *: P<0.05, n = 3. (D) Western blot with whole-cell lysates from ST2 cells infected with WNT7B or control (IE) retrovirus. Cells were serum-starved for 16 hours and then treated with inhibitor or vehicle for 2 hours before harvest. (E) Western blot of total cell lysates from ST2 cells infected with lentivirus expressing shRNA for β-catenin or LacZ, followed by retroviral infection of WNT7B or GFP (IE). (F) Western blot of total cell lysates from serum-starved ST2 cells treated with WNT3A or vehicle (V) for 1 hour with or without inhibitors (with 1-hr pretreatment). Rapa: rapamycin. (G) Western blot of total cell lysates from serum-starved ST2 cells treated with WNT3A or vehicle (V) for 1 hour with or without DKK1 (with 1-hr pretreatment). (H–I) Effects of LRP5 and/or LRP6 knockdown with shRNA. ST2 cells infected with lentiviruses were serum-starved before WNT3A treatment for 1 hour. (H): representative Western blots; (I): quantification of pS6/S6 from three independent experiments, *: p<0.05. (J) Effects of GSK3 inhibition. Serum-starved ST2 cells were treated with WNT3A for 1 hour in the presence of LiCl or NaCl.

We next investigated the signaling mechanism mediating WNT7B regulation of osteoblast differentiation. To explore the potential that WNT7B activated β-catenin signaling in bone, we used the TOPGAL transgene as a reporter in vivo [40]. By comparing the LacZ staining signal on sections of long bones from *ColI-Wnt7b* mice versus littermate controls, we did not detect any consistent upregulation of the signal in the perichondrium, trabecular or cortical bone, all tissues targeted by *ColI-Cre* (Fig. S9). We next utilized ST2 cells, a bone marrow stromal cell line undergoing osteoblast differentiation in response to virally expressed WNT7B [25]. Consistent with the in vivo finding above and our previous results, WNT7B did not activate the Lef-luciferase reporter, a readout for β-catenin signaling, in transient transfection assays [25] (Fig. 6A). However, WNT7B activated mTORC1 signaling in ST2 cells, as indicated by increased phosphorylation of S6 and 4EBP1 (Fig. 6B) (Fig. S10A). We further found that S6 and 4EBP1 phosphorylation was stimulated in the long bones of *Osx-Wnt7b* mice over the control (Fig. 6C) (Fig. S10B). Thus, WNT7B activates mTORC1 both in vitro and in vivo.

We then explored the molecular mechanism mediating mTORC1 activation by WNT. Inhibition of either PI3K or PI3K-mediated AKT activation markedly suppressed mTORC1 activity with or without WNT7B expression in ST2 cells (Fig. 6B, D), but knockdown of β-catenin had no effect (Fig. 6E). Similarly, purified recombinant WNT3A protein activated S6 and 4EBP1 phosphorylation in a PI3K- and AKT-dependent manner (Fig. 6F) (Fig. S10C). The phosphorylation of S6 is specific to mTORC1 activation as we previously showed that knockdown of raptor abolished the induction by WNT3A, and here rapamycin eliminated the phosphorylation [29] (Fig. 6F). Because the purified protein offers the advantage of studying signaling events after short-term treatments, we used WNT3A for subsequent experiments. Recombinant DKK1 protein dose-dependently suppressed WNT3A-induced mTORC1 activation (Fig. 6G and data not shown). Knockdown of LRP5 increased basal mTORC1 due to an unknown mechanism, but did not suppress the induction by WNT3A (Fig. 6H, I). In contrast, knockdown of LRP6 either alone, or together with LRP5, abolished WNT3A-induced mTORC1, indicating a predominant role of LRP6 in this regulation (Fig. 6H, I). Inhibition of GSK3 by LiCl suppressed the basal mTORC1 level, but did not reduce the extent of induction by WNT3A (Fig. 6J). Thus, WNT3A activates mTORC1 through LRP6-PI3K-AKT signaling, likely independent of GSK3 inhibition.

### WNT7B stimulates bone formation in part through mTORC1

**Figure 7:**
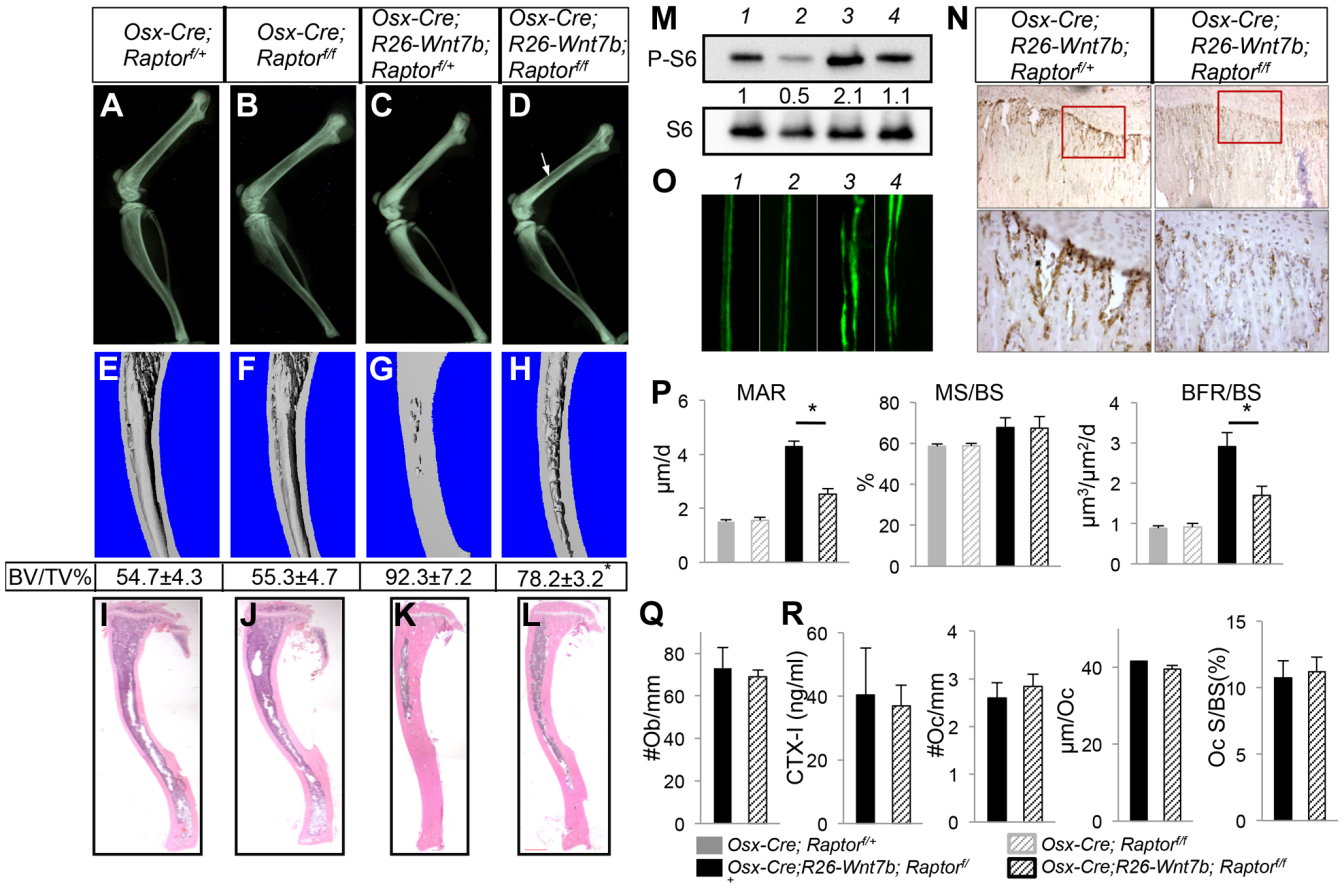
Removal of *Raptor* partially rescues WNT7B-induced bone formation. All data from mice treated with Dox from E1.5 till one month of age, then weaned off Dox for three weeks immediately before harvest. (A–D) X-ray images of hindlimbs from *Osx-Cre;Raptor^f/+^* (A), *Osx-Cre;Raptor^f/f^* (B), *Osx-Cre;R26-Wnt7b;Raptor^f/+^* (C), and *Osx-Cre;R26-Wnt7b;Raptor^f/f^* mice (D). (E–H) µCT 3D reconstruction of tibias from *Osx-Cre;Raptor^f/+^* (E), *Osx-Cre;Raptor^f/f^* (F), *Osx-Cre;R26-Wnt7b;Raptor^f/+^* (G), and *Osx-Cre;R26-Wnt7b;Raptor^f/f^* mice (H). Shown below is mean ± STDEV of combined cortical and trabecular bone volume normalized to tissue volume (BV/TV%) from three mice of each genotype. See Experimental Procedures for details. *: p<0.05 between G and H. (I–L) H&E staining of longitudinal tibia sections from *Osx-Cre;Raptor^f/+^* (I), *Osx-Cre;Raptor^f/f^* (J), *Osx-Cre;R26-Wnt7b;Raptor^f/+^* (K) and *Osx-Cre;R26-Wnt7b;Raptor^f/f^* mice (L). (M) Western blot analysis of bone extracts from *Osx-Cre;Raptor^f/+^* (lane 1), *Osx-Cre;Raptor^f/f^* (lane 2), *Osx-Cre;R26-Wnt7b;Raptor^f/+^* (lane 3), and *Osx-Cre;R26-Wnt7b;Raptor^f/f^* mice (lane 4). (N) P-S6 immunohistochemistry on longitudinal sections of tibias from *Osx-Cre;R26-Wnt7b;Raptor^f/+^* (left) and *Osx-Cre;R26-Wnt7b;Raptor^f/f^* mice (right). Shown below are images of a higher magnification for boxed areas (junction between growth plate and primary spongiosa). Signal in brown. (O) Representative images of calcein double labeling in tibias of *Osx-Cre;Raptor^f/+^* (1), *Osx-Cre;Raptor^f/f^* (2), *Osx-Cre;R26-Wnt7b;Raptor^f/+^* (3) and *Osx-Cre;R26-Wnt7b;Raptor^f/f^* mice (4). (P) Bone formation parameters from the primary ossification center. (Q) Number of osteoblasts normalized to trabecular bone perimeter (#Ob/mm) on tibia sections. (R) Osteoclast parameters. All bar graphs show mean ± STDEV, *: P<0.05, n = 3.

We next examined the potential role of mTORC1 in WNT-induced osteoblast differentiation. Rapamycin, a potent mTORC1 inhibitor, suppressed WNT7B-induced osteoblast differentiation in ST2 cells, as determined by alkaline phosphatase activity assay and von Kossa staining (Fig. S11). To test the relevance of mTORC1 activation in WNT7B-induced bone formation *in vivo*, we took advantage of *Osx-Cre* that can be suppressed by Dox to activate *R26-Wnt7b* or delete *Raptor* alone or in combination, specifically after one month of age. When *Osx-Cre* was Dox-suppressed until one month of age and then released for one month via Dox removal, the *Osx-Wnt7b* mice exhibited a profound high-bone-mass phenotype as indicated by both X-ray radiography, histology and µCT analyses (Fig. S12A, B) (Table S1). Serum biochemistry and histomorphometry confirmed that the high bone mass was caused by increased bone formation (Fig. S12C–G). In contrast, when *Osx-Cre*;*Raptor^f/f^* mice were Dox-treated till one month of age and then weaned off Dox for three weeks immediately before harvest, they did not exhibit any bone phenotype detectable by X-ray radiography, µCT or histology, when compared to either *Osx-Cre*;*Raptor^f/+^* or wild-type littermates (Fig. 7A, B, E, F, I, J and data not shown). Thus, inducible overexpression of WNT7B at one month of age caused high bone mass, but inducible deletion of *Raptor* at this age for three weeks did not affect bone mass.

Next, we asked whether deletion of *Raptor* would affect the high-bone-mass phenotype caused by WNT7B expression. To increase the ratio of the desired genotype (*Osx-Cre;R26-Wnt7b;Raptor^f/f^*) among the progenies, we set up mating pairs between *Osx-Cre; R26-Wnt7b; Raptor^f/+^* and *Raptor^f/f^* mice. Progenies with either *Osx-Cre;R26-Wnt7b;Raptor^f/^* ^+^, or *Osx-Cre;R26-Wnt7b;Raptor^f/f^* (hereafter *Osx-Wnt7b-RaptorCKO*) genotype were treated with Dox from conception until one month of age, and then weaned off Dox for three weeks before harvest. Mice with the genotype of *Osx-Cre;R26-Wnt7b;Raptor^f/+^*exhibited a very high bone mass according to X-ray radiography and µCT analyses (Fig. 7C, G). In comparison, the bone mass in the *Osx-Wnt7b-RaptorCKO* mice was notably reduced (Fig. 7D, H). Histology showed that the bone marrow cavity was expanded in the *Osx-Wnt7b-RaptorCKO* mice compared to *Osx-Cre;R26-Wnt7b;Raptor^f/+^* littermates, although still smaller than that in the *Osx-Cre*;*Raptor^f/+^*or *Osx-Cre*;*Raptor^f/f^* mice (Fig. 7I–L). Western analyses of bone protein extracts revealed that S6 phosphorylation was reduced by ∼50% in *Osx-Wnt7b-RaptorCKO* mice compared to *Osx-Cre;R26-Wnt7b;Raptor^f/+^* littermates (Fig. 7M, lanes 3 and 4). Immunohistochemistry confirmed a marked reduction of S6 phosphorylation in the primary spongiosa of *Osx-Wnt7b-RaptorCKO* mice compared to the *Osx-Cre;R26-Wnt7b;Raptor^f/+^* control (Fig. 7N). Histomorphometric studies indicated that *Raptor* deletion reduced the WNT7B-induced osteoblast hyperactivity (Fig. 7O, P), but did not suppress the increase in osteoblast number (Fig. 7Q). Moreover, *Raptor* deletion had no effect on bone resorption, as neither the serum CTX-I level nor any of the osteoclast parameters changed (Fig. 7R). Thus, mTORC1 signaling contributes to WNT7B-induced bone formation through stimulation of osteoblast function.

## Discussion

We have provided evidence that WNT7B is a potent bone anabolic protein both during embryogenesis and in the postnatal life of mice. Specifically, WNT7B markedly increases both the number and function of osteoblasts. We further identify mTORC1 as an important mediator for WNT-mediated bone anabolism. At the mechanistic level, WNT proteins activate mTORC1 through PI3K-AKT signaling.

Of note, mTORC1 appears to mediate the increase in osteoblast activity but not number in response to WNT7B. In our genetic experiments, inducible deletion of Raptor did not completely abolish S6 phophorylation induced by WNT7B in bone protein extracts. Therefore, the observed degree of correction in osteoblast activity may be an underestiamte of the full contribution of mTORC1 to WNT7B-induced osteoblast function. Because of the same reason, we cannot rule out the possibiltiy that the remaining portion of WNT7B-induced mTORC1 activtiy contributed to the increase in osteoblast number in the compound mutants. Alternatively, mTORC2 hyperactivation may be a contributing factor as we observed heightened mTORC2 signaling in the bones of the *Osx-Wnt7b* mice (data not shown). Moreover, since WNT7B also activates PKCδ through phosphoinositide signaling [25], PKCδ activation may contribute to WNT7B-induced osteoblastogenesis. On the other hand, our data do not support β-catenin as a main effector for WNT7B function in the present setting. First, WNT7B did not activate β-catenin signaling in ST2 cells although it induced osteoblast differentiation. Second, *in vivo* studies with the *TOPGAL* allele failed to detect increased β-catenin signaling in the bones of either *ColI-Wnt7b* or *Osx-Wnt7b* embryos. Finally, the bone phenotype of the *Osx-Wnt7b* mouse was distinct from that of the mouse with a stabilized form of β-catenin expressed in Osx-lineage cells, which included premature mineralization and suppression of *OC* expression [21]. Overall, a comprehensive understanding of the mechanisms underlying the potent bone anabolic function of WNT7B may provide molecular targets for developing novel bone anabolic drugs.

In addition to the strong bone anabolic effect, WNT7B also appeared to suppress osteoclast numbers when normalized to the bone surface area. This finding held true both in mice beginning to express WNT7B in the embryo (*ColI-Wnt7b*) and in those expressing it only postnatally (*Runx2-Wnt7b* with Dox). In either model, total bone resorption activity as measured by serum CTX-1 was either increased or not changed depending on the age, when compared to control littermates. Thus, we conclude that the effect of WNT7B on osteoclasts did not add to the high-bone-mass phenotype. Nonetheless, it is of future interest to determine the mechanism for the suppression of osteoclast number by WNT7B.

We show that GSK3 inhibition suppresses basal level phosphorylation of S6 but not its induction by WNT3A. This observation contradicts a previous report that GSK3 inhibition mediates mTORC1 activation by WNT3A [27], but is in agreement with another study identifying GSK3 as an activator of S6K1 via direct phosphorylation [41]. The basis for the discrepancy between these studies is not known at present. Nonetheless, our results support an alternative model that WNT proteins activate mTORC1 through PI3K-AKT signaling.

Previous studies have implicated other WNT proteins in controling bone mass. *Wnt10b^−/−^* mice showed an initial increase in bone mass at one-month of age, but subsequently exhibited age-dependent bone loss [35], [37]. Transgenic mice overexpressing WNT10B from either *FABP4* or *OC* promoter increased bone mass in postnal mice [35], [36]. However, the WNT10B-induced bone phenotype was less severe than that of the WNT7B-expressing mice. In addition, haploinsufficiency of WNT5A was reported to reduce bone mass in postnatal mice, and WNT5A was shown to stimulate both osteoblast differentiation via the suppression of PPARG-mediated adipogenesis, and osteoclastogenesis through upregulation of RANK in the macrophage progenitors [26], [39]. However, overexpression of WNT5A in our study did not have an obvious effect on bone mass. We acknowledge that our small sample size is not sufficiently powered to detect minor changes. Moreover, WNT5A may have effects on bone formation and resorption that offset each other in the overexpression model. Nonetheless, the present study identifies WNT7B as a potent anabolic WNT ligand in the mouse.

It is of interest to note that despite its robust bone anabolic activity, WNT7B did not obviously increase the width of the long bones. This observation is somewhat surprising because SOST-deficient or LRP5 high-bone-mass mutant mice displayed a clear increase in periosteal growth [15], [42]. It is possible that the SOST and LRP5 regulate endogenous WNT ligands that are of distinct signaling properties from WNT7B, or that the level of WNT7B expressed from the Rosa26 locus in our model does not reach the necessary threshold within the periosteal compartment. On the other hand, we cannot rule out the possibility that mutations in SOST or LRP5 may alter the activity of other non-WNT signals responsible for periosteal growth. Future studies are necessary to distinguish these possibilities.

## Methods

### Ethics statement

The Animal Studies Committee at Washington University has reviewed and approved all mouse procedures used in this study.

### Mouse strains

To generate the *Runx2-rtTA* transgene, we modified a *Runx2* BAC (bacterial artificial chromosome, clone# RP23-180J20) (Children's Hospital of Oakland Research Institute) to replace the first exon of *Runx2* with the cDNA for rtTA2^S^-M2 [43]. Briefly, a ∼500 bp PCR amplicon immediately upstream of the *Runx2* starting ATG (forward primer: 5′ GGAAGCCACAGTGGTAGG 3′; reverse primer: 5′ TGTAAATACTGCTTGCAGCC 3′), the cDNA for rtTA2^S^-M2 excised from pUHrT62-1 [43], and a ∼600 bp PCR amplicon immediately downstream of the *Runx2* starting ATG (forward primer: 5′ CCGTGTCAGCAAAGCTTC 3′; reverse primer: 5′ CAGGCTAATAGAGATATCTG 3′) were inserted into pSV-Flp at the PmeI, XhoI, and SalI site, respectively. The resulted plasmid was digested with AscI/PmeI to release the targeting construct. Subsequent BAC recombineering was performed as described [44], [45], [46]. Pronuclear injection was performed at Washington University Pathology/Immunology Micro-Injection Core.

The *Rosa26-Wnt7b* and *-Wnt5a* mouse strains were generated with a similar strategy as previously described for *Rosa26-ΔNGli2* [47]. The *2.3ColI-Cre*, *Osx-Cre*, *TetO-Cre*, *Wnt1-Cre*, *R26-mT/mG*, and *Raptor^f/f^* mice are as previously described [21], [48], [49], [50], [51], [52].

### Doxycycline treatment

Mice were exposed to doxycycline (Sigma, St. Louis) through drinking water containing 2% sucrose. Either 1 mg/ml or 50 µg/ml Dox in the drinking water was used for the *Runx2-rtTA* or the *Osx-Cre* mice, respectively.

### Analyses of embryonic skeleton

Whole-mount skeletal staining with alizarin red and alcian blue is as previously described [53]. For paraffin sections, dissected limbs were fixed with 10% formalin and sectioned at 6 µm thickness. For frozen sections, limbs were fixed with 4% paraformaldehyde, incubated in 30% sucrose and sectioned in OCT at 8 µm thickness. Limbs from E16.5 and older embryos were decalcified in 14% EDTA for 1–2 days after fixation. Histology and in situ hybridization with ^35^S-labeled probes were performed on paraffin sections as previously described [18], [53].

### Analyses of postnatal skeleton

X-ray radiography was performed with a Faxitron X-ray system set at 25 kv for 20 seconds. µCT analyses were performed with Scanco µCT 40 (Scanco Medical AG) according to ASBMR guidelines [54]. Quantification of the trabecular bone in the tibia was performed with 100 µCT slices (1.6 mm total) immediately below the growth plate. In the *Raptor* deletion experiment, the combined trabecular and cortical bone mass was quantified with 550 µCT slices (8.8 mm total) starting from 1.6 mm below the articular surface.

For sections, bones were fixed in 10% buffered formalin overnight at room temperature, followed by decalcification in 14% EDTA with daily change of solution for 2 weeks. After decalcification, bones were processed for paraffin embedding and then sectioned at 6 µm thickness. H&E and TRAP staining were performed on paraffin sections following the standard protocols. For dynamic histomorphometry, mice were injected intraperitoneally with calcein (20 mg/kg, Sigma, St. Louis, MO) at 7 and 2 days before sacrifice, and bones were fixed in 70% ethanol and embedded in methyl-methacrylate for plastic sections. Both static and dynamic histomorphometry were performed with the commercial software Bioquant II.

For serum-based biochemical assays, serum was collected from mice after 6 hours of fasting. Serum osteocalcin levels were determined with the Mouse Osteocalcin EIA Kit (Biomedical Technologies, Stoughton, MA). Serum CTX-I assay was performed using the RatLaps ELISA kit (Immunodiagnostic Systems, Ltd.).

Bone protein extracts were prepared from femurs and tibias of postnatal mice with RIPA buffer. The ends of the bones were surgically removed, and the bone marrow was discarded by centrifugation. The bones were then rinsed twice with cold PBS, flash-frozen in liquid nitrogen, and ground manually into a fine power with a mortar and a pestle. The bone power was incubated with 200 µl RIPA buffer on ice for 30 minutes before the supernatant was collected for Western analysis.

### Immunohistochemistry

GFP was examined either directly by fluorescence microscopy or by immunostaining on frozen sections using a chicken polyclonal GFP antibody (Abcam, Cambridge, MA). CD31 immunostaining was performed on frozen sections using a rat CD31 antibody (BD Biosciences, San Jose, CA). To detect P-S6, paraffin sections were de-paraffinized, treated with trypsin for 10 minutes, and blocked with 10% sheep serum before being incubated with a rabbit polyclonal antibody against Phospho-S6 Ribosomal Protein (Ser240/244) (Cell Signaling Technology, Danvers, MA).

### Cell culture, transfection and infection

ST2 cells were cultured in α-MEM (Sigma) with 10% fetal bovine serum (referred as growth medium). Retrovirus expressing GFP or WNT7B was produced as previously described [25], and diluted 1∶1 with growth medium before use. For viral infections, cells were incubated with the virus for 8 hours before switched to growth medium. For Western analyses of P-S6 in the virally infected cells, the cells were cultured in complete medium for 32 hours, and then in serum-free medium for 16 hours before harvest. AP staining was performed at 3 days after the viral infection. Von Kossa staining was performed with infected cells cultured for 6 days (media changed every three days) in growth medium supplemented with 50 µg/ml ascorbic acid and 10 mM β-glycerophosphate. Rapamycin (LC Laboratories) dissolved in DMSO was used at 20 nM.

For transient transfection assays, ST2 cells seeded in 24-well plate at 3×10^4^/well overnight were transfected for 8 hours with 200 ng *Lef1-luc* reporter and 20 ng *pRL-Renilla* (Promega) mixed with 1 µl Lipofectamine (Invitrogen), and then cultured in fresh growth medium for 16 hours. The transfected cells were then infected with the GFP- or WNT7B-expressing virus for 8 hours, incubated with fresh growth medium containing either vehicle or 50 ng/ml WNT3A for 2 days before harvest. Luciferase assays were performed with Dual-Luciferase Reporter Assay System (Promega).

### Antibodies, proteins and chemicals

Antibodies for S6K1, P-S6K1(T389), S6, P-S6(S240/244), FoxO3a, pFoxO1(T24)/3a(T32), P-Lrp6 (S1490), Lrp5, β-actin, and α-tubulin were purchased from Cell Signaling (Beverly, MA). Antibodies for Lrp6 and β-catenin were from Santa Cruz Biotechnology (Santa Cruz, CA).

Recombinant mouse Wnt3a and Dkk1 were purchased from R&D Systems (Minneapolis, MN), and used at 50 ng/ml and 500 ng/ml, respectively. AKT inhibitor IV was from EMD Millipore (Billerica, MA), and used at 10 µM. PI3K inhibitor LY294002 was from XXXX and used at 50 µM. LiCl and NaCl were purchased from Sigma (Saint Louis, MO) and used at 20 mM. Rapamycin was purchased from LC Laboratories (Woburn, MA), and used at 20 nM.

### shRNA knockdown

To generate shRNA lentiviruses, shRNA vectors were co-transfected into HEK293T cells with the packaging plasmids pCMV-dR8.2 dvpr (Addgene) and pCMV-VSV-G (Addgene) using FuGENE 6 (Roche). Supernatants were collected 48 hrs after transfection, and passed through 0.45 µm nitrocellulose filters. ST2 cells were infected with viral supernatants diluted 1∶1 with growth medium and supplemented with 5 µg/mL Polybrene. For the β-catenin knockdown experiment, ST2 cells were infected with shβ-catenin or shLacZ lentivirus for 8 hrs. After 16 hrs of recovery, the cells were further infected with retroviruses expressing GFP (IE) or Wnt7b (7B) for 8 hrs. After 24 hrs of recovery, the cells were then cultured in serum-free growth medium for 16 hrs before cells were lysed for Western blot. For Lrp5/6 knockdown experiment, ST2 cells were infected with shLrp5, shLrp6 or shLacZ virus for 8 hrs. Infected ST2 cells were incubated with fresh growth medium for 24 hrs, and then cultured in serum-free medium for 16 hrs. The serum-starved cells were treated with either vehicle or Wnt3a for 1 hr before being harvested for Western blot analysis.

### Statistical analyses

All quantitative data are presented as mean ± STDEV with a minimum of three independent samples. Statistical significance is determined by two-tailed Student's *t*-test.

## Supporting Information

Figure S1 Representative Southern blot of *EcoR*V-digested genomic DNA from ES cells showing correct targeting of the *Rosa26* locus. Wild-type allele: 11 kb; targeted allele: 3.8 kb. Lane 1: wild type ES cells; lane 2: ES cells carrying one *Rosa26-Wnt7b* allele.
(TIF)

Figure S2 X-ray radiography of hindlimbs from *Osx-Cre* versus *Osx-Wnt7b* mice at two months of age.
(TIF)

Figure S3 WNT7B overexpression in bone causes splenomegaly. (A–B) Whole-mount images of isolated spleens from two-month-old control (A) or *ColI-Wnt7b* littermate mice (B). (C) Quantification of spleen weight from two-month-old littermates. Bar graphs show mean ± STDEV, *: P<0.05, n = 3.
(TIF)

Figure S4 WNT7B expression maintains high bone mass in older mice. (A, B) X-ray radiography of the hindlimbs at six (A) and nine (B) months of age. (C, D) Serum CTX-I levels. Bar graphs show mean ± STDEV, *: P<0.05, n = 3.
(TIF)

Figure S5 WNT7B does not change osteocyte density in bone. Number of osteocytes were normalized to trabecular bone areas on longitudinal tibia sections from two-month-old littermate mice. n = 3.
(TIF)

Figure S6 WNT7B enhances bone formation in the late-stage embryo. Histology and *in situ* hybridization performed on longitudinal tibial sections from E18.5 control and *ColI-Wnt7b* littermate embryos.
(TIF)

Figure S7 Runx2-rtTA targets osteoblasts and bone marrow stromal cells but not growth plate chondrocytes in postnatal mice. Shown are high-resolution fluorescent images of longitudinal tibial sections from *Runx2-rtTA;TetO-Cre;R26-mTmG* mice treated with 1 mg/ml Dox in drinking water for 15 days starting at 1 month of age. Images are taken from primary ossification center (left), secondary ossification center and growth plate (middle), and bone marrow area (right). GP: growth plate.
(TIF)

Figure S8 Runx2-rtTA targets osteoblast-lineage cells expressing alkaline phosphatase (AP). AP staining (left, blue) and GFP (middle, green) immunofluorescence of frozen sections of the tibia from *Runx2-rtTA; TetO-Cre; R26-mTmG* neonates treated with 1 mg/ml Dox from E1.5 to birth. BM: bone marrow.
(TIF)

Figure S9 WNT7B does not increase β-catenin signaling. LacZ staining of frozen sections from newborn *TOPGAL* (left) or *ColI-Wnt7b; TOPGAL* (right) mice. Cells experiencing β-catenin signaling stained blue. Note robust signal in chondrocytes and few blue cells in the perichondrial region (known to be targeted by ColI-Cre).
(TIF)

Figure S10 WNT7B and WNT3A induce phosphorylation of 4EBP1. (A) Western blot with whole-cell lysates from ST2 cells infected with WNT7B or control (IE) retrovirus. Cells were serum-starved for 16 hours before harvest. (B) Western blot analyses with bone protein extracts from two-month-old *Osx-Cre* (Ctrl) and *Osx-Wnt7b* (7B) littermate mice. (C) Western blot of total cell lysates from serum-starved ST2 cells treated with WNT3A (3A) or vehicle (V) for 1 hour.
(TIF)

Figure S11 Rapamycin inhibits Wnt-induced osteoblast differentiation. Alkaline phosphatase (AP) (top) and von Kossa staining (bottom) at 72 hours and 6 days, respectively, after retroviral infection. IE: virus expressing GFP; 7b: virus expressing Wnt7b; Rapa: rapamycin.
(TIF)

Figure S12 WNT7B overexpression in one-month-old mice stimulates bone formation. *Osx-Cre* or *Osx-Wnt7b* mice were treated with Dox from conception until one month, and then weaned off Dox for one month before harvest. (A) X-ray images. (B) H&E staining of longitudinal tibial sections. (C) Serum osteocalcin levels. (D–F) Dynamic histomorphometry parameters from secondary ossification center of the tibia. MAR: mineral apposite rate; MS/BS: mineralizing surface over bone surface; BFR/BS: bone formation rate. (G) Serum CTX-I levels. Bar graphs show mean ± STDEV, *: P<0.05, n = 3.
(TIF)

Table S1 MicroCT analyses of Osx-Wnt7b mice with Dox removal from one through two months of age.
(TIF)
