# Supplementary figures and images for: WNT7B Promotes Bone Formation in part through mTORC1
Source: PLoS Genet. 2014 Jan 30;10(1):e1004145. doi: 10.1371/journal.pgen.1004145 (PMC3907335; doi:10.1371/journal.pgen.1004145)

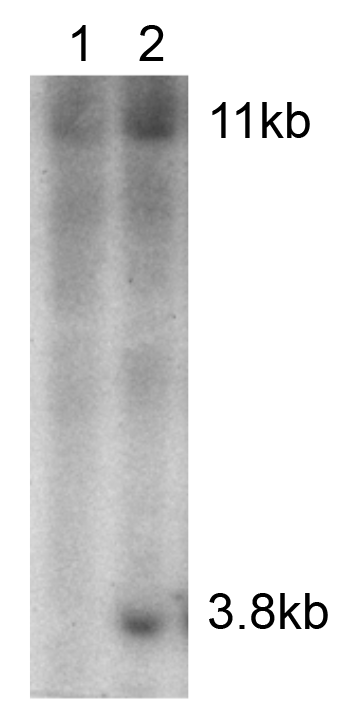

Supplement: Figure S1 — Representative Southern blot of EcoRV-digested genomic DNA from ES cells showing correct targeting of the Rosa26 locus. Wild-type allele: 11 kb; targeted allele: 3.8 kb. Lane 1: wild type ES cells; lane 2: ES cells carrying one Rosa26-Wnt7b allele. (TIF) [file pgen.1004145.s001.tif]

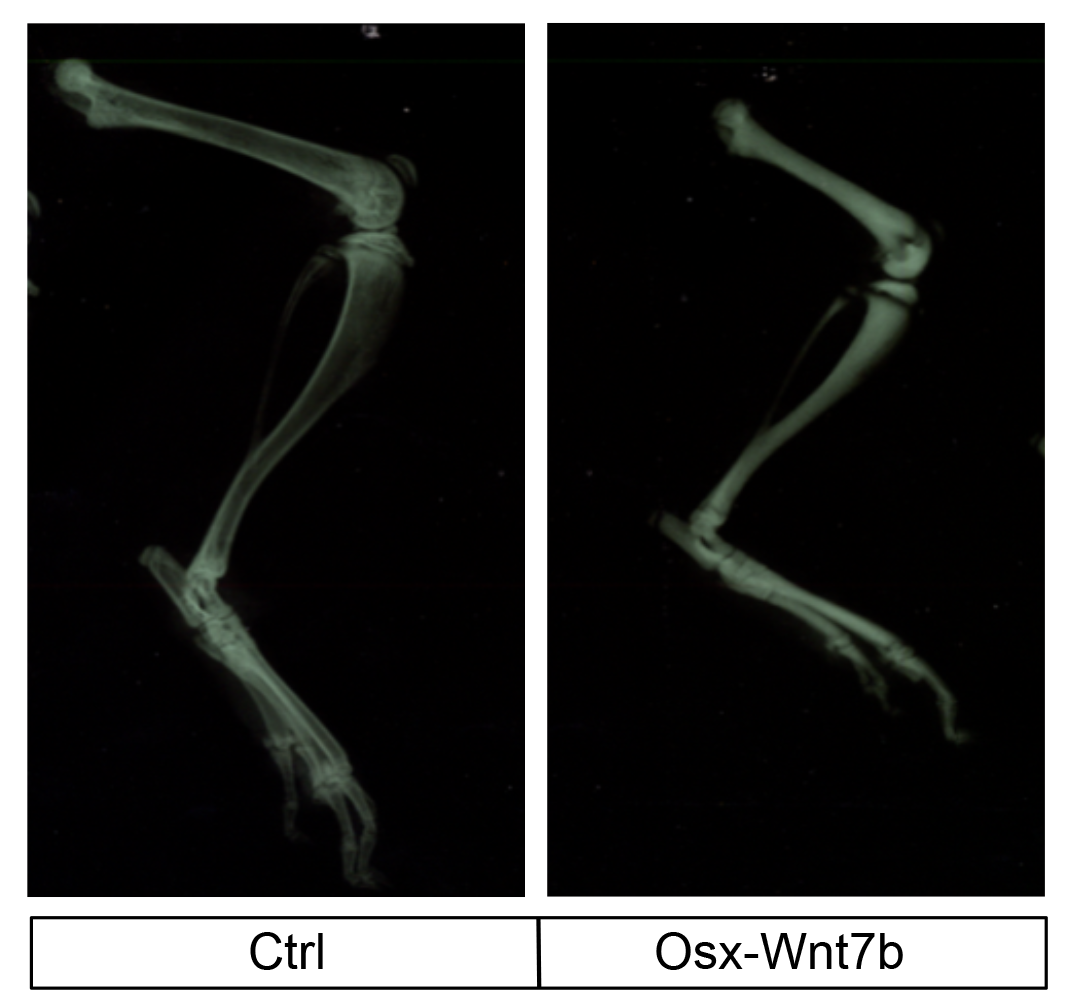

Supplement: Figure S2 — X-ray radiography of hindlimbs from Osx-Cre versus Osx-Wnt7b mice at two months of age. (TIF) [file pgen.1004145.s002.tif]

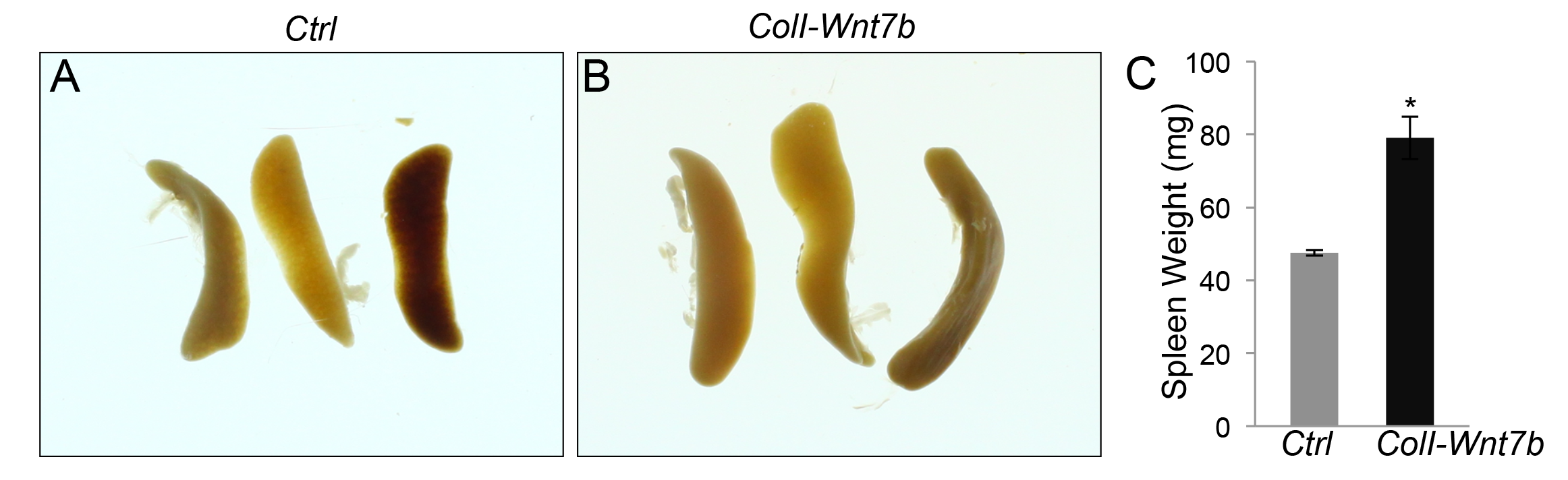

Supplement: Figure S3 — WNT7B overexpression in bone causes splenomegaly. (A–B) Whole-mount images of isolated spleens from two-month-old control (A) or ColI-Wnt7b littermate mice (B). (C) Quantification of spleen weight from two-month-old littermates. Bar graphs show mean ± STDEV, *: P<0.05, n = 3. (TIF) [file pgen.1004145.s003.tif]

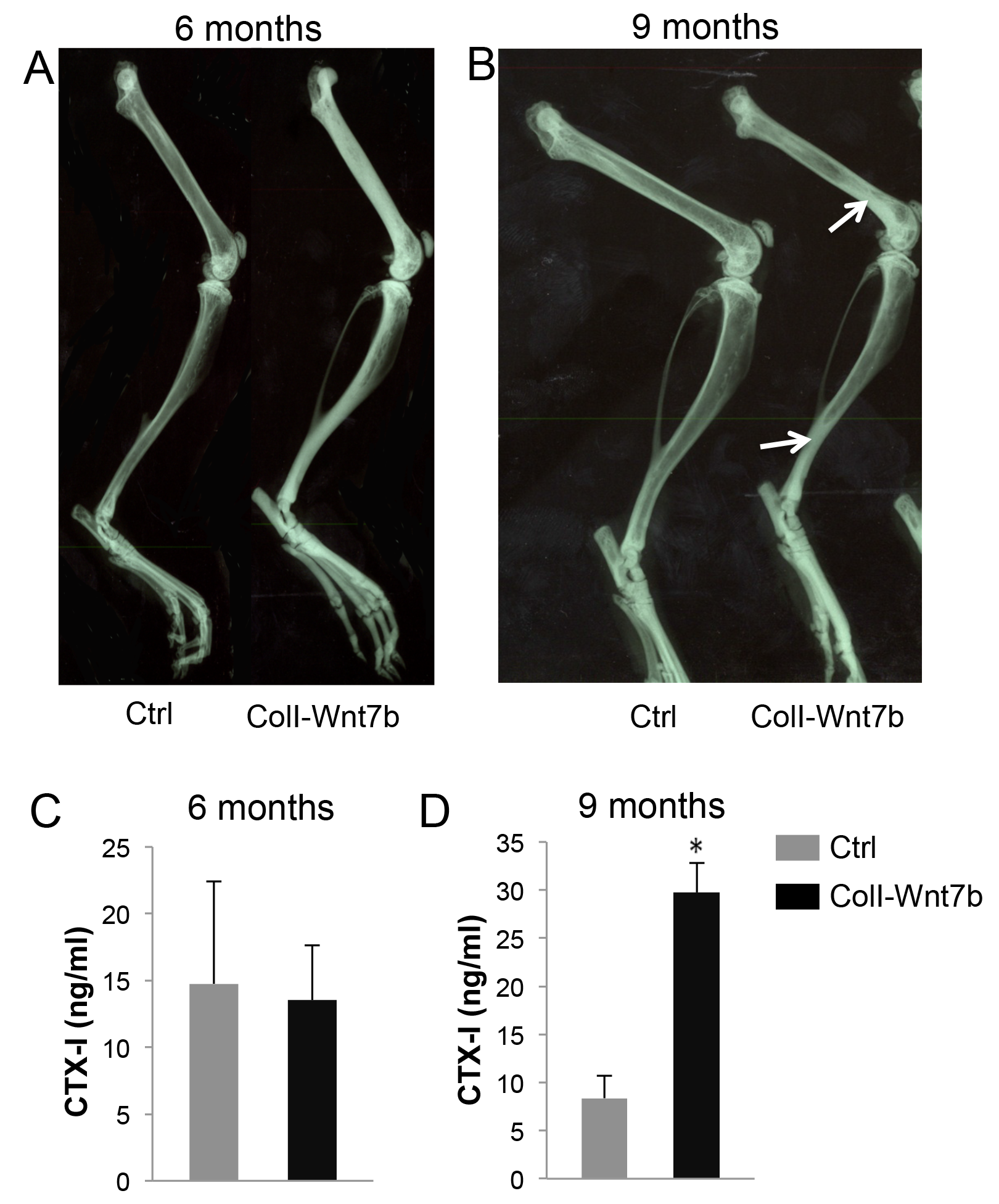

Supplement: Figure S4 — WNT7B expression maintains high bone mass in older mice. (A, B) X-ray radiography of the hindlimbs at six (A) and nine (B) months of age. (C, D) Serum CTX-I levels. Bar graphs show mean ± STDEV, *: P<0.05, n = 3. (TIF) [file pgen.1004145.s004.tif]

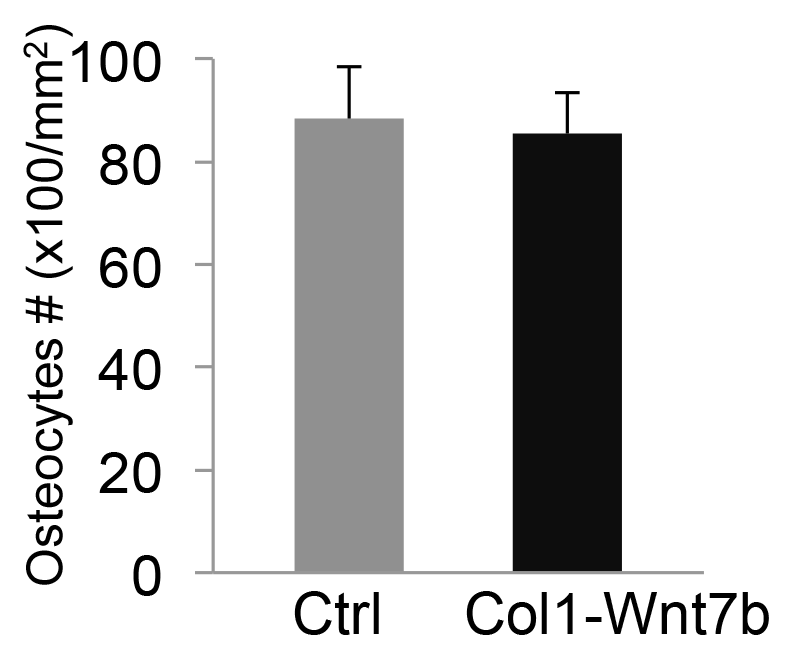

Supplement: Figure S5 — WNT7B does not change osteocyte density in bone. Number of osteocytes were normalized to trabecular bone areas on longitudinal tibia sections from two-month-old littermate mice. n = 3. (TIF) [file pgen.1004145.s005.tif]

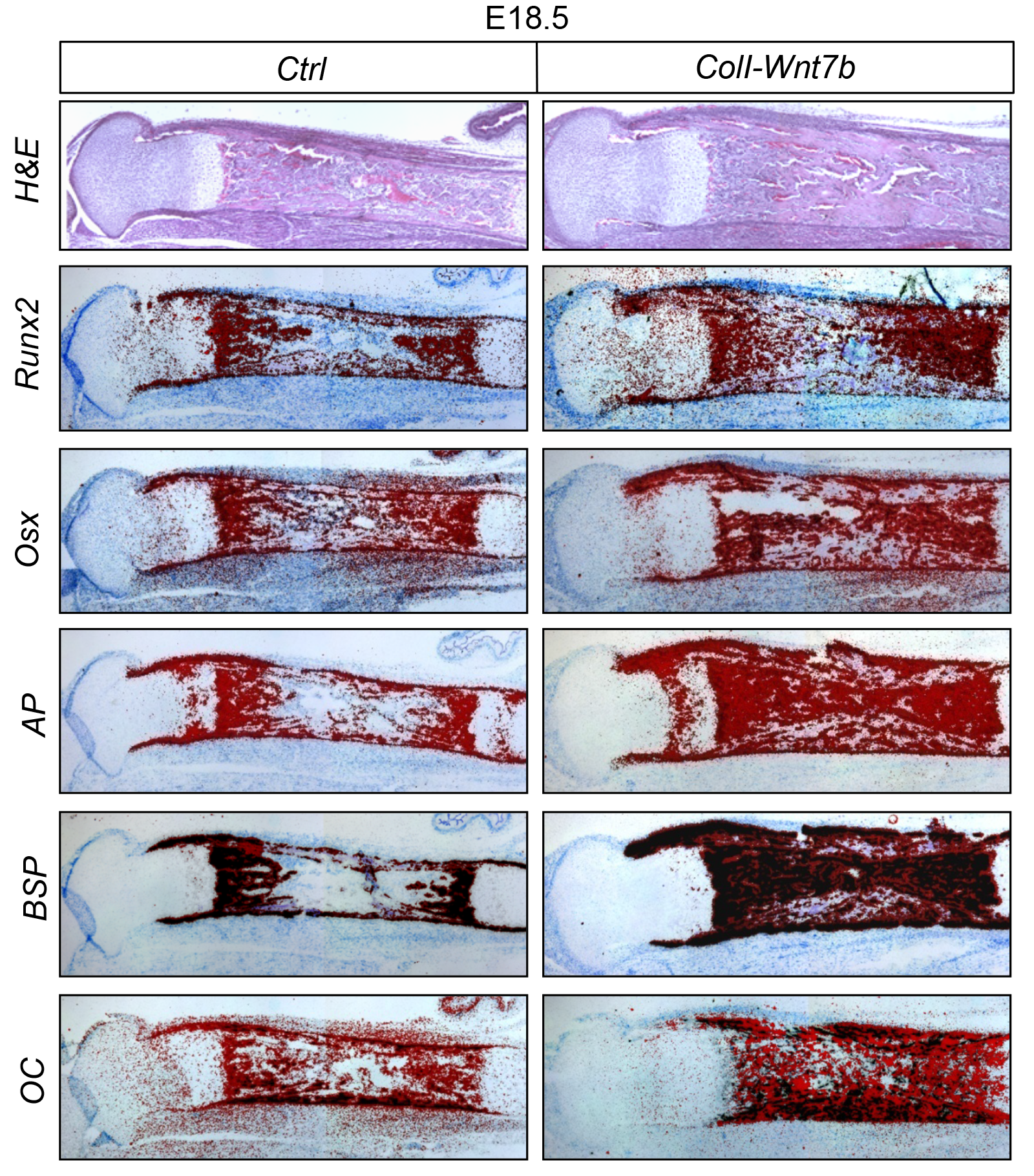

Supplement: Figure S6 — WNT7B enhances bone formation in the late-stage embryo. Histology and in situ hybridization performed on longitudinal tibial sections from E18.5 control and ColI-Wnt7b littermate embryos. (TIF) [file pgen.1004145.s006.tif]

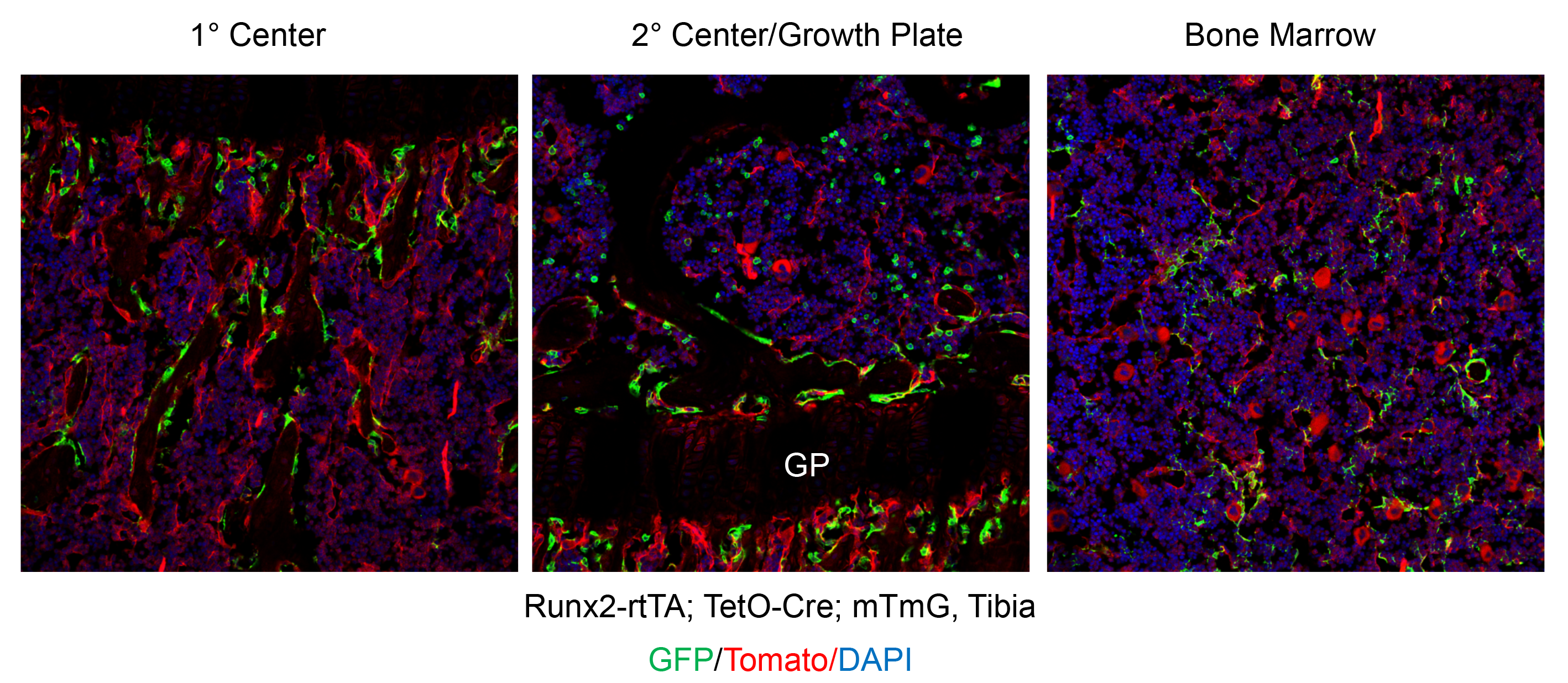

Supplement: Figure S7 — Runx2-rtTA targets osteoblasts and bone marrow stromal cells but not growth plate chondrocytes in postnatal mice. Shown are high-resolution fluorescent images of longitudinal tibial sections from Runx2-rtTA;TetO-Cre;R26-mTmG mice treated with 1 mg/ml Dox in drinking water for 15 days starting at 1 month of age. Images are taken from primary ossification center (left), secondary ossification center and growth plate (middle), and bone marrow area (right). GP: growth plate. (TIF) [file pgen.1004145.s007.tif]

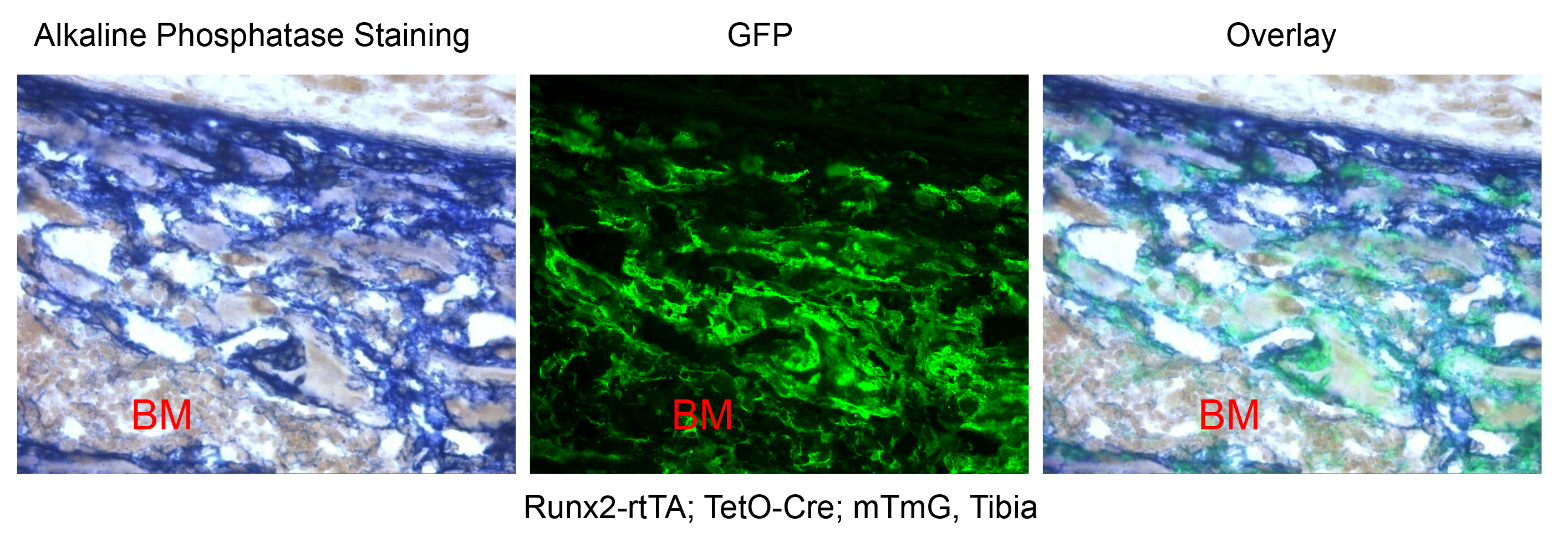

Supplement: Figure S8 — Runx2-rtTA targets osteoblast-lineage cells expressing alkaline phosphatase (AP). AP staining (left, blue) and GFP (middle, green) immunofluorescence of frozen sections of the tibia from Runx2-rtTA; TetO-Cre; R26-mTmG neonates treated with 1 mg/ml Dox from E1.5 to birth. BM: bone marrow. (TIF) [file pgen.1004145.s008.tif]

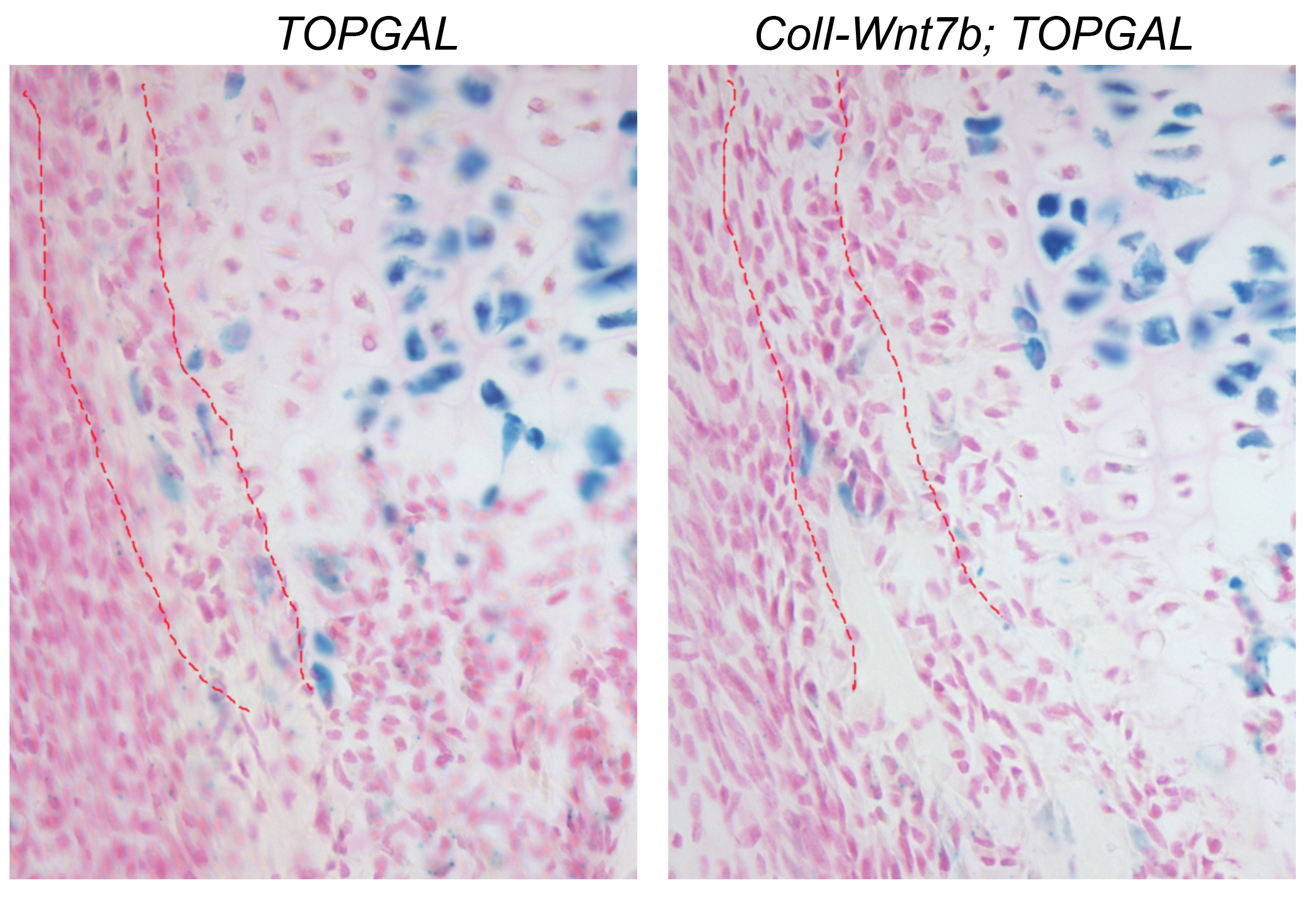

Supplement: Figure S9 — WNT7B does not increase β-catenin signaling. LacZ staining of frozen sections from newborn TOPGAL (left) or ColI-Wnt7b; TOPGAL (right) mice. Cells experiencing β-catenin signaling stained blue. Note robust signal in chondrocytes and few blue cells in the perichondrial region (known to be targeted by ColI-Cre). (TIF) [file pgen.1004145.s009.tif]

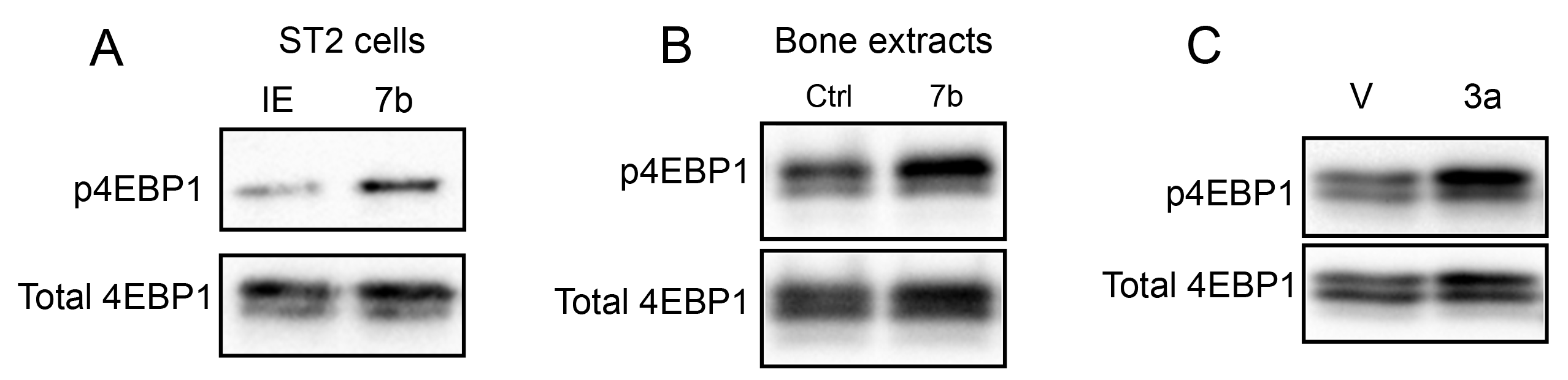

Supplement: Figure S10 — WNT7B and WNT3A induce phosphorylation of 4EBP1. (A) Western blot with whole-cell lysates from ST2 cells infected with WNT7B or control (IE) retrovirus. Cells were serum-starved for 16 hours before harvest. (B) Western blot analyses with bone protein extracts from two-month-old Osx-Cre (Ctrl) and Osx-Wnt7b (7B) littermate mice. (C) Western blot of total cell lysates from serum-starved ST2 cells treated with WNT3A (3A) or vehicle (V) for 1 hour. (TIF) [file pgen.1004145.s010.tif]

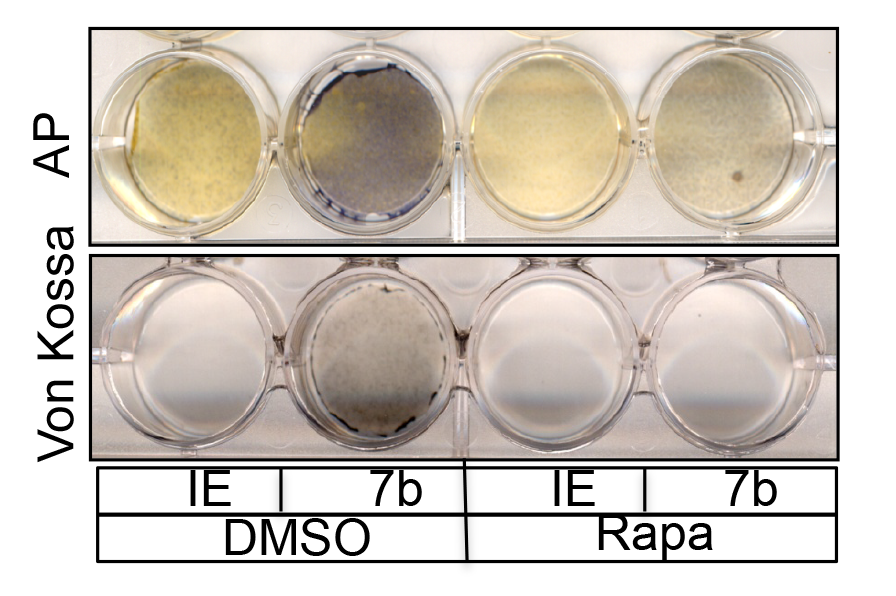

Supplement: Figure S11 — Rapamycin inhibits Wnt-induced osteoblast differentiation. Alkaline phosphatase (AP) (top) and von Kossa staining (bottom) at 72 hours and 6 days, respectively, after retroviral infection. IE: virus expressing GFP; 7b: virus expressing Wnt7b; Rapa: rapamycin. (TIF) [file pgen.1004145.s011.tif]

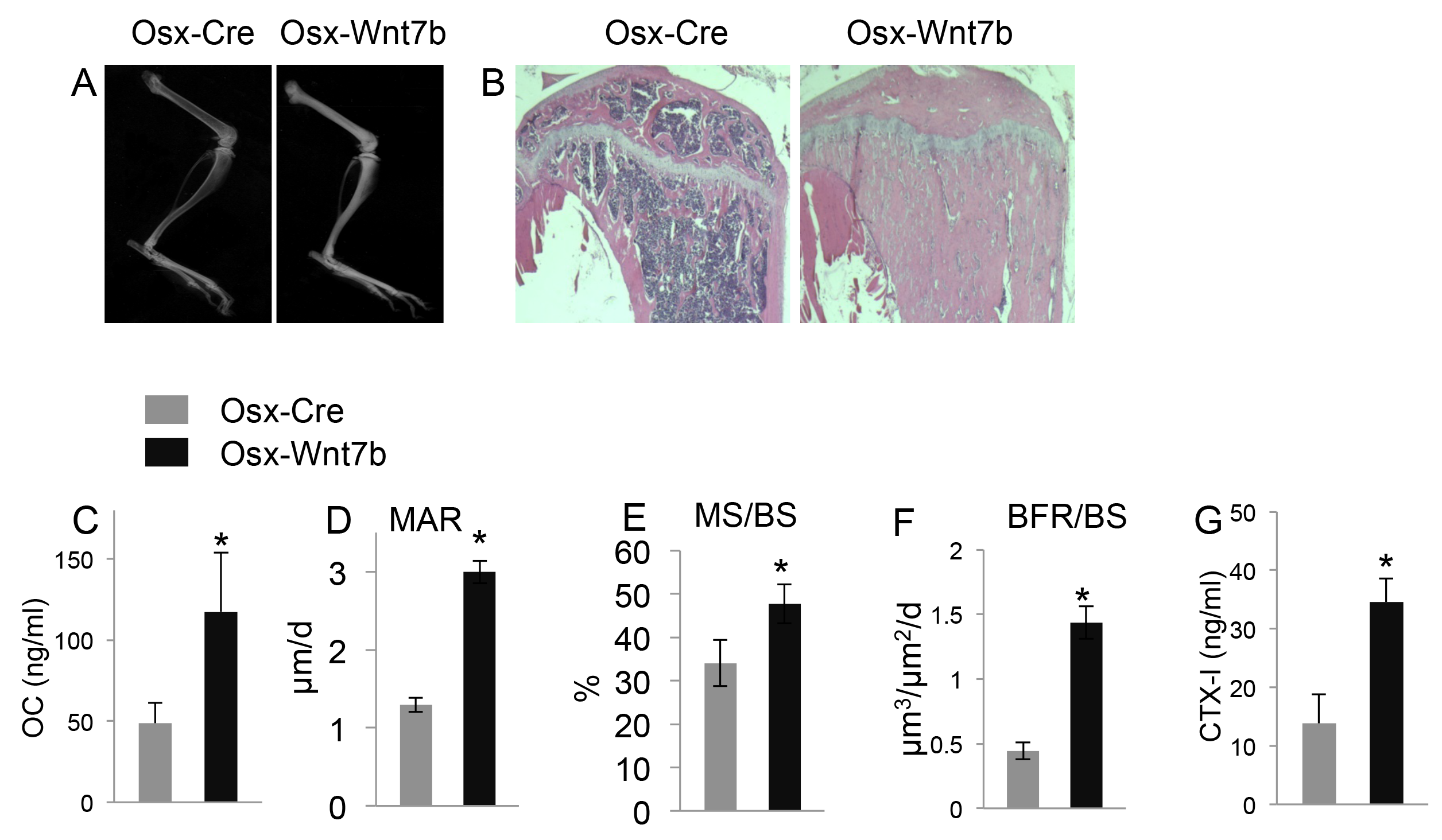

Supplement: Figure S12 — WNT7B overexpression in one-month-old mice stimulates bone formation. Osx-Cre or Osx-Wnt7b mice were treated with Dox from conception until one month, and then weaned off Dox for one month before harvest. (A) X-ray images. (B) H&E staining of longitudinal tibial sections. (C) Serum osteocalcin levels. (D–F) Dynamic histomorphometry parameters from secondary ossification center of the tibia. MAR: mineral apposite rate; MS/BS: mineralizing surface over bone surface; BFR/BS: bone formation rate. (G) Serum CTX-I levels. Bar graphs show mean ± STDEV, *: P<0.05, n = 3. (TIF) [file pgen.1004145.s012.tif]

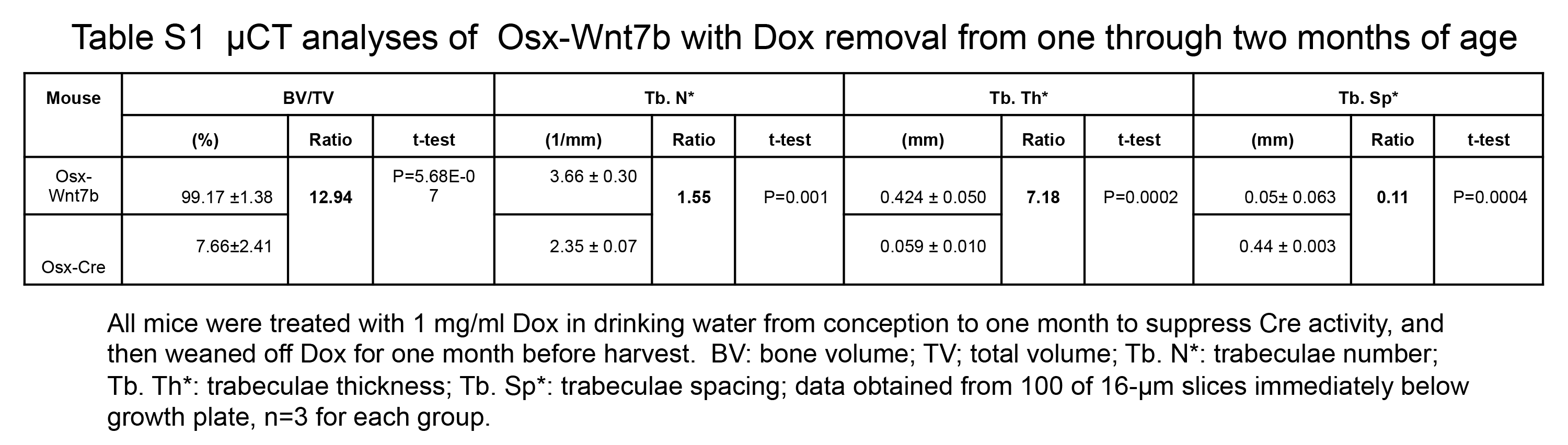

Supplement: Table S1 — MicroCT analyses of Osx-Wnt7b mice with Dox removal from one through two months of age. (TIF) [file pgen.1004145.s013.tif]
